# Dual-Mechanism Aptamer–Drug Complex Overcomes Paclitaxel Resistance in Ovarian Cancer via Structural Constraint and Telomerase Inhibition

**DOI:** 10.34133/research.1362

**Published:** 2026-07-14

**Authors:** Yuan Ma, Zefeng Chen, Xinyang Shen, Feng Ding, Nan Liu, Sifan Yu, Jia-Ke Xu, Hu Li, Aiping Lu, Huarui Zhang, Chuanxin Zhong, Yihao Zhang, Fangfei Li, Dong-Hua Yang, Tao Tang, Bao-Ting Zhang, Ge Zhang

**Affiliations:** ^1^ School of Chinese Medicine, Faculty of Medicine, The Chinese University of Hong Kong, Hong Kong 999077, China.; ^2^ Increasepharm & Hong Kong Baptist University Joint Centre for Nucleic Acid Drug Discovery, Hong Kong 999077, China.; ^3^ Law Sau Fai Institute for Advancing Translational Medicine in Bone & Joint Diseases, School of Chinese Medicine, Hong Kong Baptist University, Hong Kong 999077, China.; ^4^ Department of Obstetrics and Gynecology, Nanfang Hospital, Southern Medical University, Guangzhou 510515, China.; ^5^ Shenzhen Institutes of Advanced Technology, Chinese Academy of Sciences, Shenzhen 518055, China.; ^6^ Department of Gynecology, The First Affiliated Hospital of Jinan University, Guangzhou 510630, China.; ^7^ New York College of Traditional Chinese Medicine, Mineola, NY 11501, USA.; ^8^ Department of Obstetrics & Gynaecology, Faculty of Medicine, The Chinese University of Hong Kong, Hong Kong 999077, China.

## Abstract

Paclitaxel resistance and poor tumor selectivity remain significant challenges in epithelial ovarian cancer therapy. To overcome these challenges, we engineered PSaA360, a structurally constrained aptamer–drug conjugate with a dual-functional molecular lock that simultaneously rigidifies the AS1411 aptamer and delivers potent telomerase inhibition. Unlike the conformational flexibility of conventional aptamer–drug conjugates, PSaA360 employs the G-quadruplex stabilizer 360A to simultaneously rigidify the AS1411 aptamer into a high-affinity conformation and deliver potent telomerase inhibition. This structure-constrained and therapy-integrated strategy improved nucleolin binding, enhanced cellular internalization, and counteracted paclitaxel chemoresistance. In vivo, PSaA360 exhibited marked tumor inhibition with minimal systemic toxicity. By transforming a therapeutic agent into a structural stabilizer, PSaA360 establishes a new paradigm for mechanism-guided aptamer engineering in chemotherapy-resistant malignancies.

## Introduction

Epithelial ovarian cancer remains one of the most lethal gynecologic malignancies worldwide, accounting for a disproportionate number of cancer-related deaths. Despite breakthroughs in surgical cytoreduction and platinum–taxane chemotherapy, the 5-year survival rate for advanced disease remains low. A major obstacle to curative treatment was the rapid development of intrinsic and acquired resistance to first-line agents, particularly paclitaxel (PTX), coupled with its inherently poor water solubility and lack of tumor-selective biodistribution [1,2]. Pharmacological inhibition of telomerase has been shown to restore chemosensitivity [3]. A combination of epigallocatechin gallate and sulforaphane has been shown to trigger apoptosis in PTX-resistant ovarian cancer cells by concurrently down-regulating human telomerase reverse transcriptase (TERT) and Bcl-2 [4]. However, the pleiotropic nature of these natural products complicates the attribution of the antiresistance effect to human TERT versus Bcl-2. Thus, a focused investigation of the independent role of TERT in PTX resistance is warranted.

Aptamers are typically selected using SELEX (systematic evolution of ligands by exponential enrichment)-based technology for their target-binding properties [5]. In targeted therapy, approaches based on aptamers, including therapeutic aptamers [6–8], aptamer–drug conjugates [9–11], and aptamer-directed protein degraders [12–14], have recently arisen as a promising new frontier. Recently, Ding et al. [15] reported that radiolabeled aptamers exhibited a favorable safety profile, with no obvious uptake in normal organs, in a first-in-human clinical trial. Additionally, they developed a PTK7-targeting aptamer–monomethyl auristatin E conjugate, Sgc8c-M, which demonstrated robust antitumor activity across multiple solid tumor models in mice and exhibited a favorable safety profile in rats and macaques [16]. The G-quadruplex (G4) DNA aptamer AS1411 has emerged as a promising tumor-targeting carrier owing to its high affinity for nucleolin (NCL), a cell surface receptor markedly overexpressed on malignant ovarian cells but largely absent from normal tissues [17]. Under physiological conditions, the aptamers show important conformational flexibility and are rapidly degraded by serum nucleases, leading to reduced binding affinity, lower cellular uptake, and increased off-target effects [18]. These persistent biophysical challenges required structural engineering to lock the aptamer into a stable, high-affinity conformation.

The modulation of G-quadruplex secondary structure allows for the programming of aptamer–protein complexation kinetics [19]. Building on the conceptual success of covalent stapling strategies [20], we developed a new approach that reduced aptamer heterogeneity and improved stability. We designed PSaA360 (360A iodide-constrained AS1411–PTX complex), a structurally constrained aptamer–drug complex that used a therapeutic small molecule as a functional molecular lock. This design integrated the G4-stabilizing and telomerase-inhibiting small molecule 360A [21] into an AS1411–PTX scaffold. Unlike conventional small-molecule conjugates, 360A served a dual purpose: it structurally stabilized the aptamer while therapeutically overcoming PTX resistance. This dual-action design directly addressed 2 key barriers in ovarian cancer treatment: drug resistance and tumor selectivity. Recently, Geng et al. [22,23] demonstrated in a dual-recognition composite adsorbent the concept of using aptamers in an integrated platform to enhance specificity and structural stability, which is conceptually similar to our strategy of using 360A as a molecular lock to stabilize AS1411 aptamers to improve therapeutic function. Structurally, 360A locked AS1411 into a highly rigid G4 conformation, as confirmed by thermal melting and circular dichroism (CD) analyses. Molecular dynamics (MD) simulations provided atomic-level evidence of the design success, showing that 360A stabilized the aptamer structure, reduced conformational fluctuations, and created new protein-binding interfaces, including novel salt bridges, which markedly improved affinity and specificity for NCL. Functionally, this constrained architecture resulted in improved serum stability, enhanced cellular uptake, and preferential tumor accumulation in vivo compared to its unconstrained counterpart. At the same time, therapeutically, incorporating 360A enabled targeted codelivery of a potent telomerase inhibitor alongside PTX, effectively countering the TERT-associated resistance phenotype. As expected, PSaA360 showed superior cytotoxicity in ovarian cells and strongly suppressed malignant behaviors, including migration, invasion, and adhesion, while maintaining minimal toxicity to noncancerous ovarian epithelial cells.

In summary, PSaA360 achieved effective and safe tumor suppression in xenograft models. Its dual-mechanism action resulted in increased apoptosis and reduced proliferation. Furthermore, transcriptomic analysis showed that PSaA360 induced a strong therapeutic response by reprogramming the tumor transcriptional landscape, up-regulating oncogenic and apoptotic pathways, and modulating the tumor microenvironment. Overall, the development of PSaA360 not only produced a next-generation, highly effective chemotherapeutic platform but also established a new paradigm for mechanism-guided aptamer–drug complex engineering.

## Results

### TERT expression reversed PTX resistance in epithelial ovarian cancer

**Fig. 1.**
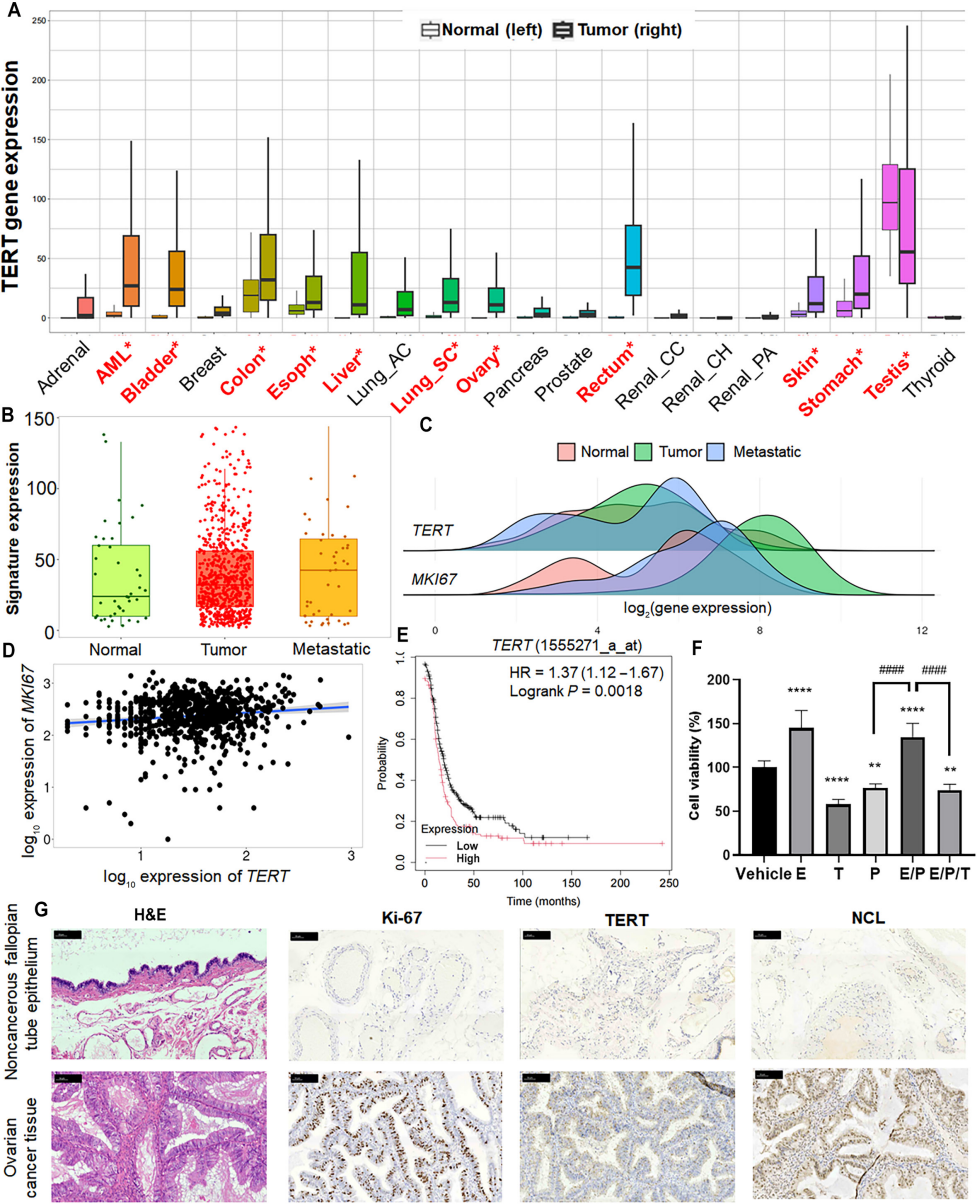
Telomerase reverse transcriptase (TERT) expression accelerates paclitaxel (PTX) resistance in epithelial ovarian cancer. (A) Pan-cancer analysis of TERT expression in normal versus tumor tissues. Red asterisks indicate significant differences (Mann–Whitney *P* < 0.05) with expression levels >10 in either group. (B) Gene signature analysis of *TERT* expression across normal, primary tumor, and metastatic tissues. (C) Coexpression analysis of *TERT* and the proliferation marker *MKI67* in normal, tumor, and metastatic tissues. (D) Correlation between *TERT* and *MKI67* expression. *n* = 744. *P* < 0.01. (E) Kaplan–Meier analysis of progression-free survival (PFS) classified by high versus low *TERT* expression. (F) The cell viability of SKOV3 cells treated with vehicle, epitalon, Telomerase-IN-3, PTX, or combination therapies, detected by Cell Counting Kit-8 (CCK-8) assay. Data represented as mean ± standard deviation (SD). *n* = 6. **P < 0.01 and ****P < 0.0001 using one-way analysis of variance (ANOVA) with Tukey’s post hoc test versus the vehicle group. ^####^*P* < 0.0001 using one-way ANOVA with Tukey’s post hoc test versus E/P group. (G) The representative immunohistochemistry (IHC) staining images of epithelial ovarian cancer tissues and noncancerous fallopian tube epithelium tissues. Scale bar: 100 μm. Vehicle, PBS (phosphate-buffered saline); E, epitalon; T, Telomerase-IN-3; P, PTX; E/P, epitalon plus PTX; E/P/T, epitalon plus PTX plus Telomerase-IN-3.

We first performed integrated bioinformatic and functional analyses to establish TERT as a functionally relevant molecular target in epithelial ovarian cancer. A pan-cancer analysis revealed TERT to be a highly dysregulated target, with pronounced overexpression across multiple tumor types, while epithelial ovarian cancer exhibited particularly high and concerning expression levels (Fig. 1A and B). Clinically, TERT overexpression in epithelial ovarian cancer showed a strong positive correlation with the marker of proliferation Ki-67 (*P* < 0.01), underscoring its central role in driving aggressive, proliferative growth (Fig. 1C and D). Survival analysis further solidified this clinical relevance: patients with high TERT expression had a markedly shorter progression-free survival (PFS; *P* = 0.0018; Fig. 1E), confirming TERT as a prognostic biomarker for poor outcomes.

To functionally validate TERT as one of the key driving factors for drug resistance, we employed pharmacological modulation. Consistent with a pro-proliferative role, the telomerase activator epitalon increased SKOV3 cell proliferation by 45%, whereas the inhibitor Telomerase-IN-3 reduced it by 42%. Most critically, epitalon actively attenuated the efficacy of PTX in SKOV3, demonstrating a TERT-driven resistance phenotype. This resistance was completely reversed when cells were cotreated with Telomerase-IN-3, indicating that TERT inhibition was sufficient to reverse PTX resistance (Fig. 1F).

In line with the in vitro results, immunohistochemistry (IHC) analysis of patient samples confirmed the co-elevation of TERT, the target receptor NCL, and the proliferation marker Ki-67 in cancerous tissues compared to noncancerous fallopian tube epithelium tissues (Fig. 1G). Collectively, these findings establish TERT as a critical molecular target for overcoming PTX resistance in epithelial ovarian cancer.

### The structurally constrained AS1411–PTX complex (PSaA360) was prepared and characterized

To address the limitations of poor stability and conformational flexibility in traditional aptamer complexes, we developed a structural engineering strategy. This involved incorporating the therapeutic telomerase inhibitor 360A as a functional molecular lock into the AS1411–PTX scaffold, resulting in the final complex PSaA360. Prior to synthesis, we confirmed the relevance of our targeting receptor NCL. Kaplan–Meier analysis verified the clinical importance of the NCL receptor, linking high *NCL* expression with a markedly shortened PFS (*P* = 0.02, Fig. S1a and b). Furthermore, receiver operating characteristic (ROC) analysis demonstrated the predictive value of NCL expression for PTX response, with an area under the curve of 0.66 in patient samples and 0.73 in cell lines (Fig. S1c and d). Flow cytometry (FCM) confirmed that AS1411 exhibited preferential and efficient specific uptake in epithelial ovarian cancer cells compared to noncancerous HEK293T cells (Fig. S1e). thereby validating the efficacy of NCL-mediated PTX delivery system for ovarian cancer treatment. Moreover, Cell Counting Kit-8 (CCK-8) analysis showed that PTX combined with 360A (PTX360) exhibited superior antiproliferative activity compared to 360A alone and PTX alone, thereby distinguishing the single-agent effect from its synergistic contribution (Fig. S1f).

**Fig. 2.**
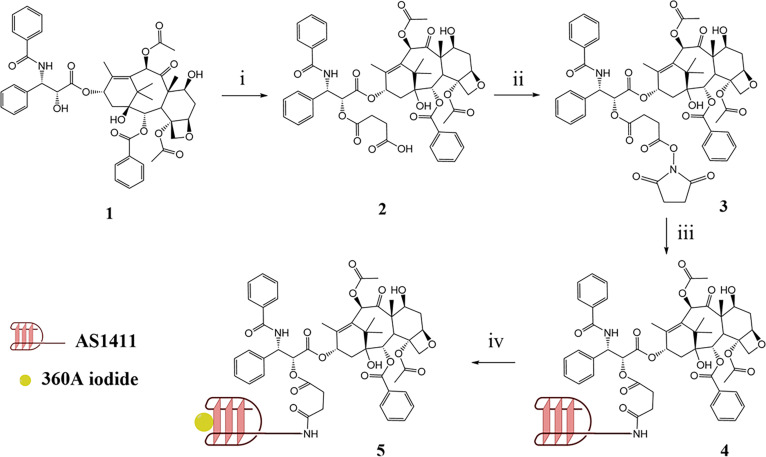
Synthetic routes of the structure-constrained AS1411–paclitaxel (PTX) complex. Reagents and conditions: (i) acetic anhydride, pyridine, and DCM; (ii) NHS, DCC, and THF; (iii) NH_2_–AS1411, NaHCO_3_ buffer (pH 8.5), and DMF; and (iv) 360A iodide and H_2_O/DMSO. DCM, dichloromethane; NHS, *N*-hydroxysuccinimide; DCC, dicyclohexylcarbodiimide; THF, tetrahydrofuran; DMF, dimethylformamide; DMSO, dimethyl sulfoxide.

Synthesis began with selective acylation of PTX’s 2′-OH using acetic anhydride and pyridine as a catalyst (Fig. 2). The resulting intermediate was then activated with *N*-hydroxysuccinimide (NHS) and *N*,*N*′-dicyclohexylcarbodiimide (DCC) to form a succinimidyl carbonate ester, which was subsequently reacted with the 5′-amino group of NH_2_–AS1411 in bicarbonate buffer (pH 8.5) to yield the core PSaA conjugate. The crucial final step involved structural constraint: the purified PSaA conjugate was incubated with 360A iodide. This reaction produced the structurally consolidated product PSaA360, which was then purified via ultrafiltration. We utilized proton nuclear magnetic resonance (^1^H NMR), carbon-13 nuclear magnetic resonance (^13^C NMR), mass spectrometry, and x-ray diffraction to fully validate the structure of all key intermediates (Figs. S2 to S7). High-performance liquid chromatography (HPLC) quantification confirmed the 1.13:1 (~1:1) stoichiometric ratio of 360A to the PSaA scaffold (Figs. S8 to S10).

### 360A-mediated structural constraint conferred superior aptamer stability and markedly enhanced NCL affinity

**Fig. 3.**
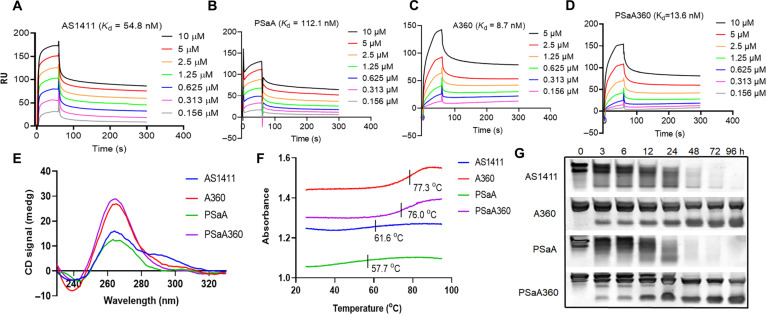
360A enhances the structural stability and nucleolin (NCL) binding of AS1411–paclitaxel (PTX). (A to D) The interactions of AS1411, PSaA, A360, and PSaA360 groups with NCL were detected by the surface plasmon resonance (SPR) assay. (E) The secondary structures of the indicated groups were detected by the circular dichroism (CD) assay. (F) The *T*_m_ values, indicating the thermodynamic stability of the AS1411, A360, PSaA, and PSaA360 groups, were detected by an ultraviolet spectrograph. (G) The serum stability of the indicated groups, detected by polyacrylamide gel electrophoresis (PAGE) assay. These samples at a concentration of 0.2 μM were incubated in 10% fetal bovine serum at 37 °C and processed at various time points. PSaA, AS1411–PTX conjugate; A360, 360A iodide-constrained AS1411 complex; PSaA360, 360A iodide-constrained AS1411–PTX complex.

We integrated 360A to address AS1411’s intrinsic biophysical instability and enhance its targeting performance. We assessed the effects of this structural constraint on both NCL-binding kinetics and aptamer stability. Surface plasmon resonance analysis was used to quantify the binding dynamics. Conventional conjugation, i.e., simple attachment of PTX to AS1411 (PSaA), might impair NCL binding (*K*_d_ = 112.1 nM versus 54.8 nM for free AS1411). Critically, the incorporation of 360A dramatically reversed this trend, translating the structural modification into superior binding affinity. A360 (360A iodide-constrained AS1411 complex) achieved a potent *K*_d_ = 8.7 nM, while the final constrained complex PSaA360 maintained high affinity (*K*_d_ = 13.6 nM), representing an 8.2-fold improvement over the unconstrained PSaA conjugate (Fig. 3A to D and Fig. S11). This enhanced binding affinity is attributable to the structural rigidification supported by 360A.

CD spectroscopy confirmed that 360A acts as a potent G4 stabilizer. PSaA360 exhibited a characteristic G4 signature with a peak nearly double that of the unconstrained AS1411 (28.9 mdeg versus 16.0 mdeg), indicating a highly organized and stable G4 structure (Fig. 3E and Table S1). Thermal melting analysis provided further thermodynamic evidence of this stabilization, showing that PSaA360 exhibited a markedly elevated melting temperature (*T*_m_ = 76.0 °C) compared to unconstrained PSaA (*T*_m_ = 57.7 °C) (Fig. 3F and Fig. S12). The structural integrity was verified through serum stability assays, which demonstrated a markedly extended half-life for PSaA360 and A360 compared to their unconstrained counterparts (Fig. 3G).

Collectively, these results established 360A as a functional structural “lock” that successfully addressed the 2 major hurdles of aptamer therapeutics: enhancing inherent stability against degradation and improving target-binding affinity.

### PSaA360 exhibited superior tumor-specific uptake and potent antitumor efficacy in vitro

**Fig. 4.**
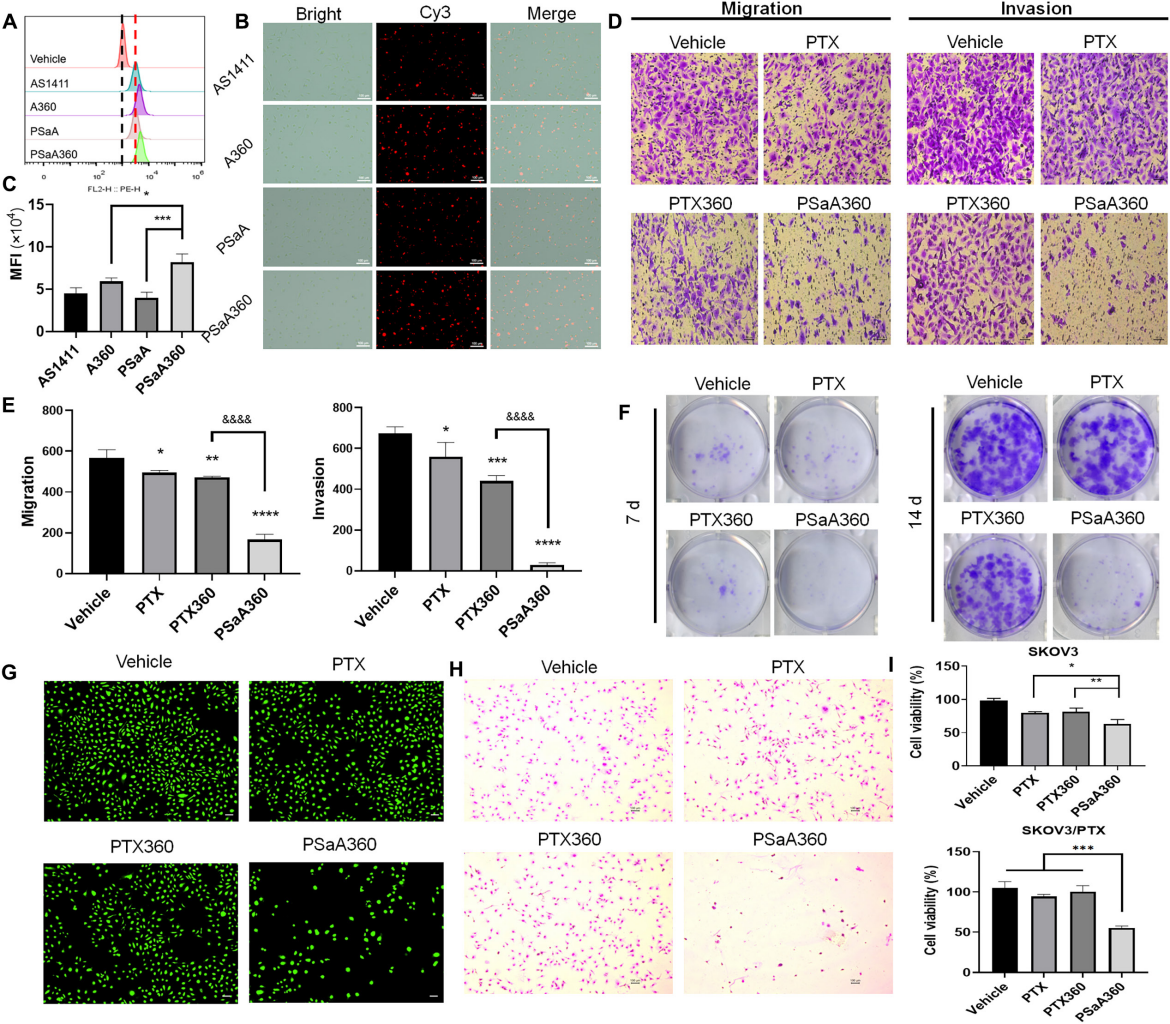
PSaA360 enhances tumor-specific uptake and anticancer ability in vitro. (A) The cellular uptakes of the vehicle, AS1411, A360, PSaA, and PSaA360 groups on SKOV3, detected by flow cytometry (FCM) assay. The representative images (B) and mean fluorescence intensity (MFI) (C) of cyanine 3 (Cy3)-labeled AS1411, A360, PSaA, and PSaA360 groups on SKOV3, detected by confocal microscopy. Scale bar: 100 μm. *P < 0.05 and ***P < 0.001 versus the PSaA360 group using one-way analysis of variance (ANOVA) with Tukey’s post hoc test. The representative images (D) indicating the migration (left), invasion (right), and statistics (E) of the vehicle, paclitaxel (PTX), PTX360, and PSaA360 groups on SKOV3, detected by transwell assays. The fixed cells were stained with crystal violet (purple). *P < 0.05, **P < 0.01, ***P < 0.001, and ****P < 0.0001 versus the vehicle group using one-way ANOVA with Tukey’s post hoc test. ^&&&&^*P* < 0.0001 versus the PSaA360 group using an unpaired 2-tailed Student *t* test. (F) The representative images indicating the colony formation of the vehicle, PTX, PTX360, and PSaA360 groups on SKOV3 cells for 7 and 14 d at 1.375 nM, detected by ImageScanner III. The fixed cells were stained by crystal violet (purple). (G) The representative images indicating live cells of the vehicle, PTX, PTX360, and PSaA360 groups on SKOV3 cells at 5.5 nM for 48 h, detected by fluorescent inverted microscope. The live cells were stained with calcein AM (green). Scale bar: 100 μm. (H) The representative images indicating adhesive cells of 48-h pre-treatment of the vehicle, PTX, PTX360, and PSaA360 groups on SKOV3 cells at 5.5 nM, detected by fluorescent inverted microscope. The fixed cells were stained with Giemsa stain (pink). (I) The inhibitory effect of the vehicle, PTX, PTX360, and PSaA360 groups on SKOV3 cells (5.5 nM) and SKOV3/PTX cells (22 nM), detected by Cell Counting Kit-8 (CCK-8) assay. Data are presented as mean ± SD. *n* = 3. *P < 0.05 and **P < 0.01 versus the PTX group using one-way ANOVA with Tukey’s post hoc test.

After confirming enhanced NCL affinity biophysically, we next examined whether 360A-mediated constraint translates into improved cellular uptake and superior in vitro antitumor activity. To evaluate uptake efficiency, we treated SKOV3 cells with various cyanine 3 (Cy3)-labeled constructs (AS1411, A360, PSaA360, and PSaA). FCM and confocal microscopy analyses demonstrated the superior internalization of the constrained complex (Fig. 4A to C). Specifically, A360 and PSaA360 exhibited a markedly higher fluorescence intensity compared with their unconstrained counterparts (AS1411 and PSaA, respectively). This compelling finding confirms that the incorporation of 360A successfully improved the tumor-specific uptake of the aptamer, likely due to the enhanced NCL affinity and structural stability previously observed. We then assessed the multiple anticancer activities of PSaA360 against SKOV3, comparing it with those of vehicle, free PTX, and PTX360 (PTX + 360A iodide). PSaA360 demonstrated profound inhibitory effects on multiple malignant behaviors: In transwell migration and wound-healing assays (Fig. 4D and E and Fig. S13), PSaA360 exhibited the lowest migratory rate among all groups. Similarly, transwell invasion assays (Fig. 4D and E) showed that PSaA360 yielded the greatest suppression of cellular invasion (*P* < 0.0001). Colony formation assays (Fig. 4F) and live-cell staining (Fig. 4G) further confirmed the compound’s potent antiproliferative effect, with PSaA360 generating the smallest number of viable colonies and live cells. Cell adhesion assays (Fig. 4H) revealed that PSaA360 obviously reduced cellular adhesion. Importantly, cytotoxicity assays (Fig. 4I and Figs. S14 and S15) revealed that PSaA360 obviously reduced viability in SKOV3 cells, SKOV3/PTX cells, A2780 cells, and A2780/PTX cells among all groups. In noncancerous IOSE-80 ovarian epithelial cells, PSaA360 induced the lowest cytotoxicity among all groups (Fig. S15), confirming that NCL-targeting effectively minimizes off-target toxicity.

Collectively, these results demonstrated that PSaA360 not only utilized the 360A-mediated structural constraint to enhance tumor-specific uptake but also exerted superior, highly targeted inhibitory effects on the hallmark characteristics of epithelial ovarian cancer (migration, invasion, proliferation, and adhesion) while maintaining an excellent safety profile on noncancerous cells.

### PSaA360 achieved potent, targeted tumor suppression without toxicity in vivo

**Fig. 5.**
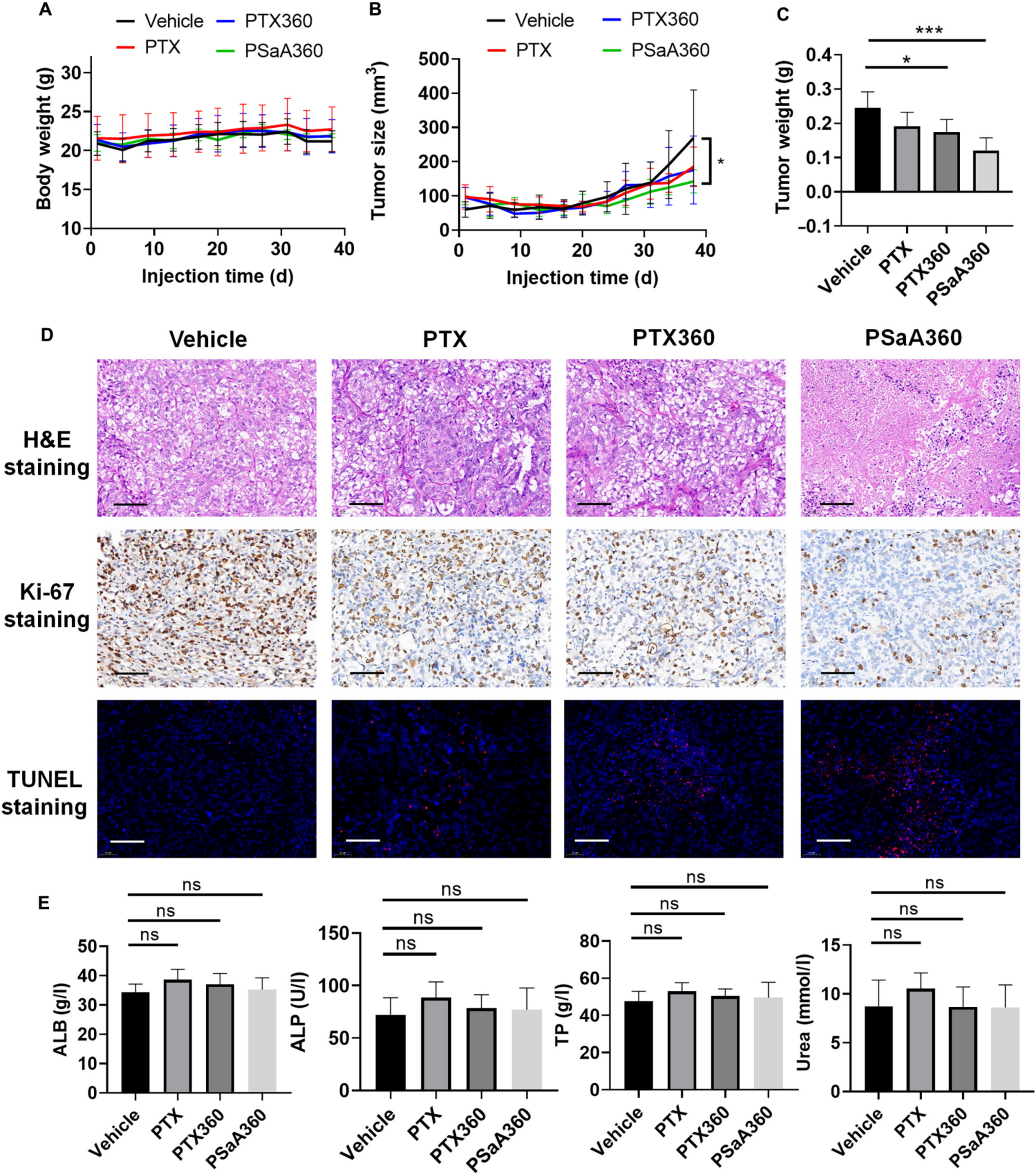
PSaA360 suppresses tumor growth without toxicity in vivo. The body weights (A) and tumor sizes (B) of the vehicle, paclitaxel (PTX), PTX360, and PSaA360 groups on the SKOV3-inoculated BALB/c nude female mice at a dosage of 5.9 μmol·kg^−1^ (with the equivalent PTX concentration of 5 mg·kg^−1^) given to mice by intravenous injection twice a week for 4 weeks. *P < 0.05 using Mann–Whitney *U* test at day 38. (C) The tumor weights of the xenografted epithelial ovarian tumors after 38 d of different treatments from the indicated groups. *P < 0.05 and ***P < 0.001 using one-way analysis of variance (ANOVA) with Tukey’s post hoc test. (D) H&E staining analysis, Ki-67 immunohistochemical staining analysis, and terminal deoxynucleotidyl transferase dUTP nick end labeling (TUNEL) immunofluorescence analysis of the xenografted epithelial ovarian tumor sections from the groups indicated. The extracellular matrix and cytoplasm were stained as pink, Ki-67-positive staining was indicated as brown, nuclei were stained as blue, and apoptosis in situ was stained as red. Scale bars, 100 μm. Results are presented as mean ± SD. *n* = 5. (E) The kidney and liver functions of the BALB/c nude mice of different treatments from the groups indicated. Data are expressed as mean ± SD. *n* = 5, followed by one-way ANOVA with Tukey’s post hoc test. ns, *P* > 0.05 versus the vehicle group. ALB, albumin; ALP, alkaline phosphatase; TP, total protein; H&E, hematoxylin and eosin.

We next evaluated the translational potential and therapeutic efficacy of PSaA360 in SKOV3 xenograft mouse models. Prior to treatment, IHC analysis confirmed the high and selective expression of NCL within the tumor tissues, while expression was rarely detected in vital organs, validating the in vivo targeting rationale (Fig. S16). Throughout the study, all treatment groups showed good systemic tolerability, as evidenced by the absence of significant differences in body weight (Fig. 5A). The PSaA360 complex yielded superior antitumor efficacy. Tumors in the PSaA360-treated group were markedly smaller than those in the free PTX group and PTX360 group (*P* < 0.05; Fig. 5B). Consistently, the final tumor weights in the PSaA360 group were markedly reduced compared with those in the vehicle control group (*P* < 0.001; Fig. 5C). Histological examination of excised tumors revealed pronounced indicators of cell death, including obvious nuclear condensation and cytoplasmic shrinkage. Furthermore, Ki-67 staining demonstrated a markedly reduced proliferative index, while terminal deoxynucleotidyl transferase dUTP nick end labeling (TUNEL) fluorescence indicated obviously enhanced apoptotic activity (Fig. 5D). Critically, the biosafety profile of PSaA360 was favorable. Routine liver function tests (including albumin, alkaline phosphatase, and total protein) and kidney function tests (urea) showed no significant differences among any of the treatment groups (*P* > 0.05; Fig. 5E). Further validation of the structural constraint’s effect was provided by biodistribution analysis, which revealed the preferential and high-level accumulation of PSaA360 specifically in tumor tissue compared with the less stable, unconstrained PSaA conjugate (Fig. S17).

Collectively, these in vivo results demonstrated that the structurally constrained PSaA360 complex translated its enhanced targeting and dual mechanism of action into potent suppression of epithelial ovarian tumor growth while maintaining a safety profile, thereby establishing its high potential as a clinical candidate.

### PSaA360 induced profound transcriptional reprogramming to modulate oncogenic and apoptotic networks

**Fig. 6.**
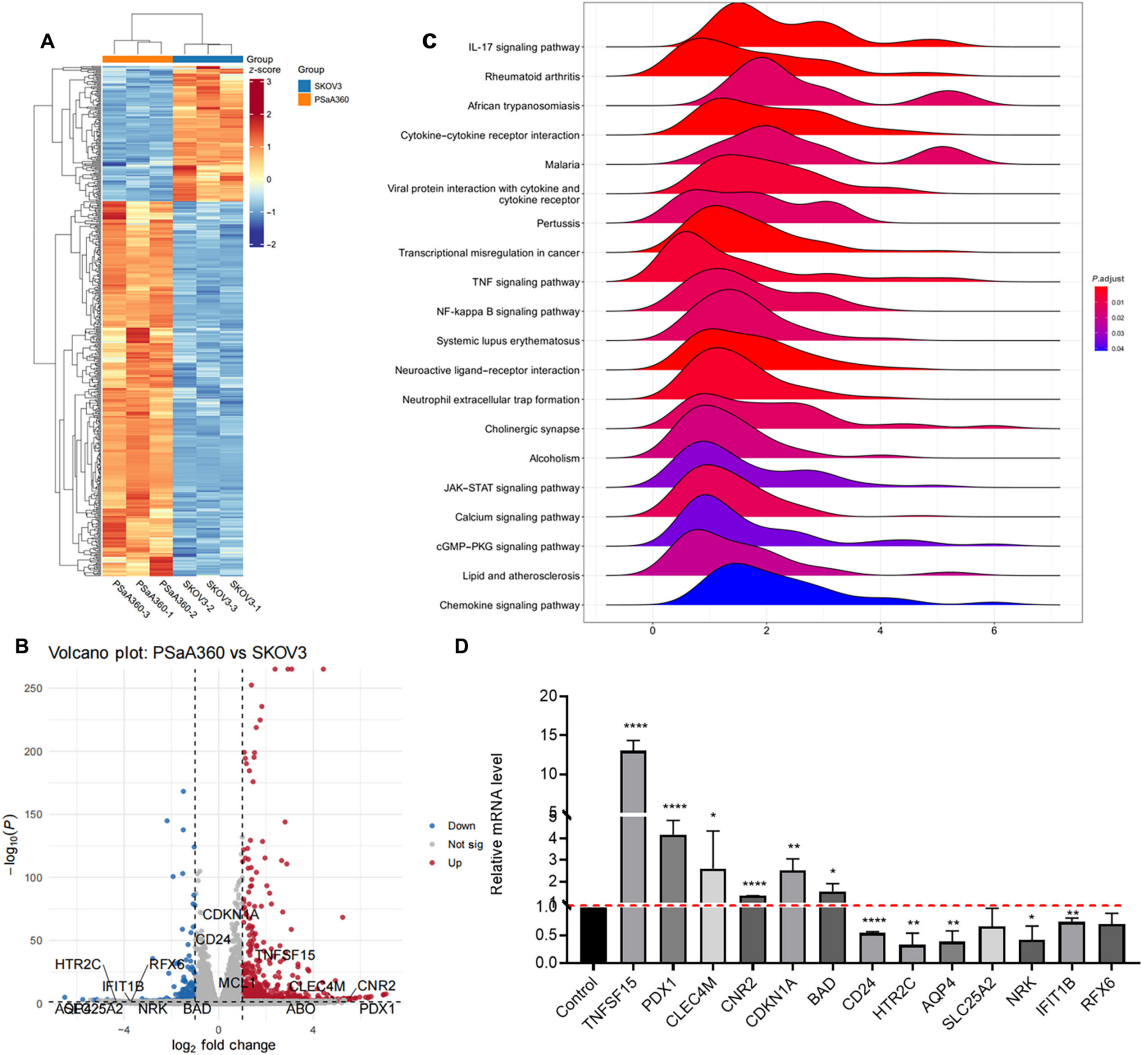
PSaA360 induces transcriptional reprogramming in epithelial ovarian cancer. (A) Heatmap showing differential gene expression between PSaA360 and SKOV3 cell control groups. (B) Volcano plot illustrating up-regulated (red) and down-regulated (blue) genes after PSaA360 exposure. (C) Kyoto Encyclopedia of Genes and Genomes (KEGG) pathway enrichment highlighting the activation of oncogenic and apoptotic signaling pathways. (D) Quantitative polymerase chain reaction (qPCR) analysis of candidate genes confirming transcriptomic data. Obvious up-regulation of *TNFSF15*, *PDX1*, *CLEC4M*, *CNR2*, *CDKN1A*, and *BAD* and down-regulation of *CD24*, *HTR2C*, *AQP4*, *NRK*, and *IFIT1B* with *P* < 0.05. Data are presented as mean ± SD. *n* = 3. *P < 0.05, **P < 0.01, and ****P < 0.0001 versus the control group using one-way analysis of variance (ANOVA) with Tukey’s post hoc test.

To uncover the molecular mechanisms underlying PSaA360’s efficacy, we performed RNA sequencing analysis on treated SKOV3 cells. This analysis identified hundreds of differentially expressed genes using stringent thresholds. Heatmap visualization (Fig. 6A) and volcano plot analysis (Fig. 6B) revealed a distinct transcriptional profile induced by PSaA360, indicating a reversal of the intrinsic oncogenic signature of the cells. Notably, we observed obvious up-regulation of several tumor-suppressive genes, including *TNFSF15* (a key mediator of apoptosis and inflammation) and *CDKN1A* (p21, a universal cell cycle inhibitor) [24]. Conversely, prosurvival oncogenes, such as *CD24* [25] and *HTR2C* [26], were markedly down-regulated.

Pathway enrichment analysis (Fig. 6C) revealed the functional consequences of this transcriptional reprogramming. We identified obvious modulation of pathways including interleukin-17 (IL-17), tumor necrosis factor (TNF), and Janus kinase/signal transducer and activator of transcription (JAK/STAT) signaling, which are known to play pleiotropic roles in cancer cells, regulating apoptosis, proliferation, and chemosensitivity independently of immune infiltration. Simultaneously, the treatment led to the repression of pathways associated with apoptosis inhibition and calcium signaling, thereby attenuating the cells’ survival capacity.

Quantitative polymerase chain reaction (qPCR) validation (Fig. 6D) confirmed the transcriptomic findings, with selected key genes showing statistically significant changes. The elevated levels of inflammation-related and differentiation-associated transcripts further supported a shift toward a pro-apoptotic phenotype induced by PSaA360. Moreover, the observed enrichment of NF-kappa B and chemokine signaling pathways suggests the potential involvement of these pathways in the cell-intrinsic response to treatment. Collectively, these results demonstrated that PSaA360 exerted its superior antitumor efficacy through multiple mechanisms involving the comprehensive reprogramming of oncogenic transcriptional networks in cancer cells. The observed modulation of key signaling pathways reflected cell-intrinsic transcriptional changes that might contribute to enhanced apoptosis and reduced chemoresistance.

### Structural constraint by 360A created novel binding interfaces with NCL

**Fig. 7.**
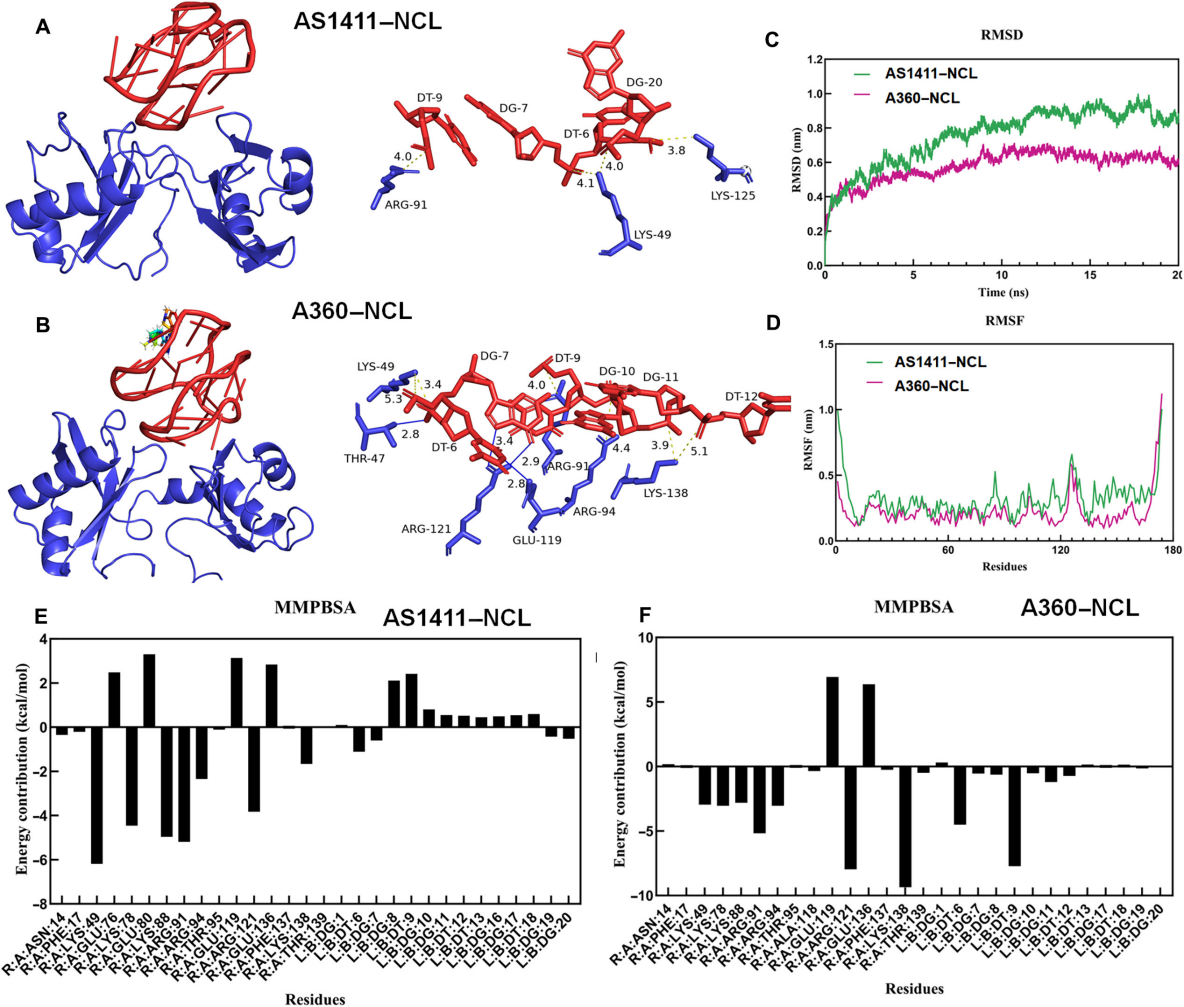
Insight into the interaction modes of structure-constrained AS1411 to nucleolin (NCL) protein. (A) The AS1411–NCL RNA-binding domain (RBD) complex structure predicted by molecular docking. (B) A360–NCL RBD complex structure predicted by molecular docking. (C) The root mean square deviation (RMSD) curves of the AS1411–NCL RBD complex and A360–NCL RBD complex evaluated by molecular dynamics (MD) simulation. (D) The root mean square fluctuation (RMSF) curves of the AS1411–NCL RBD complex and A360–NCL RBD complex evaluated by MD simulation. (E) The binding free energy for the AS1411–NCL RBD complex evaluated by the molecular mechanics Poisson–Boltzmann surface area (MMPBSA) method. (F) The binding free energy for the A360–NCL RBD complex evaluated by the MMPBSA method.

To obtain atomic-level evidence that 360A functions as a molecular lock to enhance binding, we performed MD simulations and docking studies of AS1411 and A360 constructs with NCL. The incorporation of 360A demonstrated its powerful structural stabilizing effect on the AS1411 aptamer. MD simulations revealed a marked reduction in the root mean square deviation (RMSD) (Fig. S18), root mean square fluctuation (RMSF) (Fig. S19), and total energy (Fig. S20). These reductions confirmed that 360A markedly restrained the conformational movement and heterogeneity of the aptamer, resulting in a highly stable, rigidified 3-dimensional structure. Crucially, this structural constraint was translated into a markedly enhanced interaction with the NCL target. Further analysis of the constrained A360–NCL complex revealed the pivotal role of 360A in remodeling and reinforcing aptamer–protein interactions. Specifically, the rigidified structure promoted the formation of novel binding sites between key residues in the aptamer and the NCL_RBD1/2 domain, including G10-Arg94, G11-Lys138, and T12-Lys138. As a direct consequence of this structural engineering, the total number of aptamer–NCL interaction contacted more than doubled, increasing from 4 in the unconstrained AS1411 complex to 10 in A360 complex (Fig. 7A and B and Table S2). This marked increase highlighted the enhanced binding potential conferred by structural constraint. Consistently, the RMSD and RMSF values for A360–NCL complex were lower than those for the AS1411–NCL complex (Fig. 7C and D), providing computational confirmation of improved conformational stability within the bound state. Importantly, molecular mechanics Poisson–Boltzmann surface area (MMPBSA) calculations provided mechanistic details, showing that 360A incorporation neutralized the unfavorable inhibitory effects of GLU76 and GLU80 on AS1411 binding while simultaneously strengthening highly favorable interactions involving T6 and T9 (Fig. 7E and F).

Collectively, these computational findings provided mechanistic validation for our engineering hypothesis. They demonstrated that the structural integration of 360A not only rigidly stabilized the AS1411 aptamer but also created entirely new binding interfaces, thereby markedly enhancing its affinity and specificity for NCL, a key advancement for next-generation aptamer therapeutics.

## Discussion

**Fig. 8.**
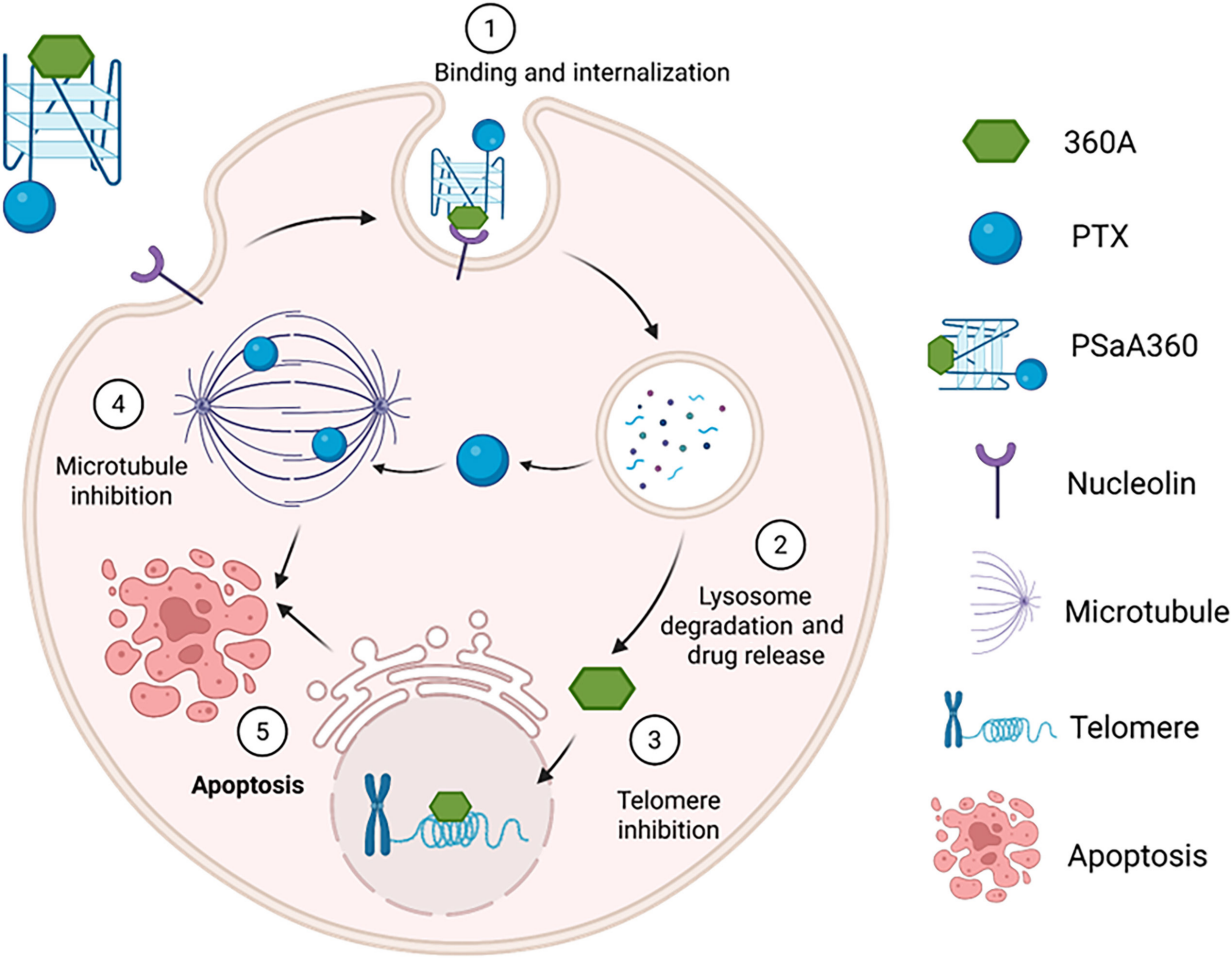
A schematic diagram illustrating the proposed mechanism of action of the PSaA360 complex. BioRender.com.

Chemoresistance remains a critical challenge in oncology, particularly in epithelial ovarian cancer, where it severely limits treatment efficacy [27]. Our clinical analysis and in vitro data confirmed that TERT expression accelerates PTX resistance in epithelial ovarian cancer. Thus, codelivery of a TERT inhibitor with PTX represents a rational strategy to treat epithelial ovarian cancer. In our previous work, we employed AS1411, a G4 DNA aptamer, for the targeted delivery of PTX to tumors [10]. However, conformational flexibility and poor serum stability remain major barriers to clinical translation [28]. Our strategy integrates 360A, a potent telomerase inhibitor and G4 stabilizer, into the AS1411–PTX scaffold. Unlike the conventional G4 stabilization strategies such as 2′-deoxyinosine and d-/l-isothymine modification [29], our approach utilizes 360A as a therapeutic activity stabilizer. This transforms the flexible aptamer into a structurally constrained, high-affinity delivery platform (Fig. 8). To our knowledge, no prior study has integrated a therapeutic G4 stabilizer as a structural molecular lock into an aptamer–drug conjugate to simultaneously enhance conformational stability, improve targeting affinity, and reverse PTX resistance. Specifically, motivated by the clinical challenge of PTX resistance and our demonstration that TERT overexpression drives this phenotype, we report the first integrated 360A–AS1411–PTX complex. Unlike prior cell-free studies that examined only pairwise interactions, our “therapeutic lock” design exploits 360A’s dual functionality, as a structural stabilizer to enhance targeting and as a telomerase inhibitor to reverse resistance. Atomic-level simulations show that 360A remodels the protein-binding interface, and in vivo validation confirms potent efficacy with excellent safety, advancing the field from biochemical observation to a resistance-reversing therapeutic platform.

The preparation of PSaA360 was achieved by simply mixing PSaA and 360A in solution. With 3 equivalents of 360A relative to PSaA, the resulting loading ratio was 0.47. When 6 equivalents of 360A were used, the loading ratio increased to 1.08. Using 9 equivalents led to only a modest increase (or no obvious increase) in the loading ratio, reaching 1.14 (Fig. S21). Thus, the final ratio of 360A to PSaA in the product is approximately 1:1. The resulting construct, PSaA360, embodies a dual mechanism: it selectively delivers PTX to NCL-overexpressing tumor cells while concurrently inhibiting telomerase activity to counteract chemoresistance. This integrated approach concurrently enhances stability, improves targeting affinity, and reverses chemoresistance, transforming a conventional aptamer–drug conjugate into a structurally constrained, dual-mechanism platform. This design yielded a dramatic increase in NCL-binding affinity and serum stability, effectively converting AS1411 into a precision-guided vehicle.

Functionally, PSaA360 demonstrated superior performance compared to conventional PTX formulations across multiple dimensions. In vitro, it potently suppressed proliferation, migration, invasion, adhesion, and colony formation in cancer cells while sparing noncancerous cells. Cell adhesion was also markedly reduced by PSaA360 relative to all other treatments. Cytotoxicity assays further showed that PSaA360 reduced viability in both parental (SKOV3 and A2780) and PTX-resistant (SKOV3/PTX and A2780/PTX) cells, confirming its ability to overcome acquired resistance. In noncancerous IOSE-80 cells, PSaA360 exhibited the lowest cytotoxicity (*P* < 0.01), confirming that NCL targeting minimizes off-target toxicity.

Transcriptomic analysis further illuminated the mechanistic basis, revealing modulation of IL-17, TNF, and JAK/STAT pathways. These pathways regulate apoptosis, proliferation, and chemosensitivity in a cell-intrinsic manner, independent of immune infiltration [30,31]. This cell-intrinsic transcriptional reprogramming aligns with the dual mechanism of PSaA360: targeted PTX delivery via NCL recognition and concurrent telomerase inhibition via 360A-mediated G-quadruplex stabilization. By counteracting TERT-driven resistance, PSaA360 reprograms signaling networks that govern survival and proliferation, linking structural design to functional outcome. In vivo, these molecular effects translated into profound tumor suppression, reduced Ki-67 proliferation indices, and enhanced apoptosis, all without observable systemic toxicity. This combination of efficacy and safety positions PSaA360 as a next-generation chemotherapeutic strategy that overcomes key limitations of conventional drug conjugates.

Mechanistic insights from docking and MD simulations provided atomic-level clarity for the observed functional gains; 360A incorporation stabilized AS1411’s conformation, reducing RMSD and RMSF, and created new binding interfaces with NCL’s RBD1/2 domain. The formation of new salt bridges and hydrogen bonds strengthened aptamer–protein interactions and neutralized inhibitory residues, offering a structural rationale for the enhanced affinity observed experimentally. These insights establish a framework for mechanism-guided engineering of future aptamer–drug complexes. Our 360A stabilization strategy enhances specificity via conformational selection, stabilizing the active parallel G4 conformation of AS1411. AS1411 exists in solution as an equilibrium of multiple conformations, with only a subset exhibiting high NCL affinity [32]. By selectively binding and stabilizing the active conformation, 360A shifts this equilibrium toward the high-affinity state without disturbing the loop structures critical for target recognition. MD simulations further support this induced fit, demonstrating that the stabilized conformation presents NCL-binding residues in an optimal spatial orientation, as reflected by the increased interaction contacts (10 versus 4 in the unconstrained AS1411 complex).

Despite promising therapeutic potential, several limitations must be addressed before clinical translation. First, 360A loading was inferred from HPLC and release assays rather than directly proven; orthogonal methods (e.g., labeled-tracer recovery and covalent cross-linking) are needed to confirm stoichiometry and ensure quality control. Second, a complete in vivo pharmacokinetic profile is lacking and should be obtained using a validated liquid chromatography–tandem mass spectrometry (LC–MS/MS) assay in rodents. Third, the current in vivo cohort size was relatively small. We acknowledge that larger cohorts would further strengthen statistical confidence, and we plan to validate our observations in expanded preclinical studies. Finally, scale-up considerations, including solid-phase synthesis for batch consistency, assessment of immunogenicity, manufacturing cost management, and regulatory planning for a combination drug/biological product, must be addressed in future development.

In conclusion, PSaA360 represents an advance in aptamer–drug conjugate design. By rationally integrating the G4 stabilizer and telomerase inhibitor 360A into the AS1411–PTX scaffold, we have created a single-agent platform that concurrently addresses the delivery (stability and targeting) and resistance (TERT inhibition) challenges that limit conventional chemotherapy. This mechanism-guided engineering, transforming a therapeutic agent into a structural stabilizer, provides a versatile blueprint for developing targeted therapies against NCL-positive, chemotherapy-resistant malignancies.

## Materials and Methods

### Materials

The DNA aptamers utilized in this research were synthesized on an ÄKTA oligopilot Plus 100 standard DNA/RNA synthesizer. Phosphoramidites were purchased from Chemvon Biotechnology Co., Ltd, while other compounds were purchased from Sigma-Aldrich. Penicillin–streptomycin was purchased from Gibco by Life Technologies, and fetal bovine serum was purchased from Thermo Fisher Scientific. Dulbecco’s modified Eagle’s medium (DMEM) was purchased from Omacgene, and the CCK-8 was purchased from MedChemExpress. Matrigel was purchased from Corning. Transwell inserts were purchased from NEST Biotechnology. Paraformaldehyde (PFA; 4%), Giemsa, and crystal violet were purchased from Beyotime. Calcein AM was purchased from Invitrogen. BALB/c nude mice were purchased from the Laboratory Animal Services Center at the Chinese University of Hong Kong.

### Clinical data analysis

Ovarian cancer cohorts from the National Center for Biotechnology Information (NCBI) Gene Expression Omnibus (https://www.ncbi.nlm.nih.gov/geo/) and the Genomic Data Commons Data Portal (https://portal.gdc.cancer.gov/) were examined, focusing on samples with transcriptome-level data from at least 10 patients. The validation of NCL and TERT expression in ovarian cancer was executed using the Kaplan–Meier plotter analysis platform (https://www.kmplot.com) [33]. Real-time comparisons of gene expression alterations between tumor tissues and normal tissues were carried out using the TNM plotter analysis platform (https://www.tnmplot.com) [34]. The TNM-plotter pipeline for data processing and analysis was developed in R version 3.6.1. Additionally, multiple transcriptome-level gene expression datasets, along with treatment and response data, were integrated into a unified database. Linking NCL expression with PTX response in ovarian cancer patients and cells was investigated using transcriptome-level data on the ROC plotter analysis platform (https://rocplot.com/ovarian/index) [35]. Responder and nonresponder patients were compared using 2 distinct statistical methodologies.

### Synthesis of the AS1411–PTX conjugate

PTX (7.761 g, 9.088 mmol) was dissolved in anhydrous dichloromethane (DCM; 400 ml) and reacted with acetic anhydride (1.31 g, 11.1 mmol) and anhydrous pyridine (2.7 ml, 33.5 mmol) at room temperature for 3 d. Following the reaction, the mixture was purified using silica gel chromatography (DCM/methanol = 20/1 to 3/1) to yield compound 2 as a white powder (8.7 g). Then, compound 2 (3.7017 g, 3.880 mmol), NHS (451.4 mg, 3.925 mmol), and DCC (831.1 mg, 4.028 mmol) were dissolved in 120 ml of anhydrous tetrahydrofuran. After stirring at room temperature for 3 d, the mixture underwent recrystallization in pre-cooled ether (500 ml) at 4 °C. The crude compound 3 (4.1995 g) was obtained. Next, compound 3 was dissolved in dimethylformamide (50 mM, 120 μl) along with a solution of NaHCO_3_ (50 mM, 60 μl) and added to NH_2_–AS1411 (100 nmol). The reaction proceeded at 30 °C for 2 h and was quenched by the addition of a 2 M solution of triethylammonium acetate (TEAA; 100 μl, pH = 7.28). Finally, AS1411–PTX was purified and desalted using Agilent 1260 Semi-Prep LC System and characterized by LC–MS.

Compound 2: MS (electrospray ionization [ESI]) *m*/*z* for C_51_H_55_NO_17_Na^+^ [M + Na]^+^, calculated 976.3362, found 976.3376; ^1^H NMR (400 MHz, deuterated methanol [MeOD]) δ 8.13 to 8.09 (m, 2H), 7.81 (dd, *J* = 5.2, 3.4 Hz, 2H), 7.67 (t, *J* = 7.4 Hz, 1H), 7.58 (t, *J* = 7.5 Hz, 2H), 7.55 to 7.41 (m, 7H), 7.26 (t, *J* = 7.2 Hz, 1H), 6.44 (s, 1H), 6.06 (s, 1H), 5.83 (d, *J* = 6.3 Hz, 1H), 5.63 (d, *J* = 7.2 Hz, 1H), 5.48 (d, *J* = 6.4 Hz, 1H), 5.02 to 4.95 (m, 1H), 4.33 (dd, *J* = 10.9, 6.6 Hz, 1H), 4.18 (s, 2H), 3.80 (d, *J* = 7.2 Hz, 1H), 2.72 (dd, *J* = 7.3, 5.3 Hz, 2H), 2.65 to 2.59 (m, 2H), 2.46 (ddd, *J* = 14.5, 9.6, 6.6 Hz, 1H), 2.38 (s, 3H), 2.25 to 2.10 (m, 5H), 1.91 (d, *J* = 0.7 Hz, 3H), 1.86 to 1.75 (m, 2H), 1.64 (s, 3H), 1.13 (d, *J* = 4.8 Hz, 6H); ^13^C NMR (101 MHz, MeOD) δ 205.2 (s), 175.8 (s), 173.5 (s), 171.6 (s), 171.3 (s), 170.5 (d, *J* = 18.7 Hz), 167.7 (s), 142.5 (s), 138.4 (s), 135.6 (s), 134.7 (d, *J* = 18.1 Hz), 132.9 (s), 131.3 (d, *J* = 17.3 Hz), 130.1 (s), 129.9 to 129.5 (m), 128.7 (d, *J* = 1.6 Hz), 85.9 (s), 82.3 (s), 79.1 (s), 77.5 (s), 76.9 (s), 76.3 (s), 76.0 (s), 73.0 (s), 72.3 (s), 59.2 (s), 55.3 (s), 47.9 (s), 44.6 (s), 37.5 (s), 36.5 (s), 29.7 (d, *J* = 17.1 Hz), 27.0 (s), 23.3 (s), 22.4 (s), 20.8 (s), 15.0 (s), 10.5 (s). Peak overlapping was observed. Compound 3: MS (ESI) *m*/*z* for C_55_H_59_N_2_O_19_^+^ [M + H]^+^, calculated 1,051.3707, found 1,051.3690.

### Crystal structure analysis

Approximately 20 mg of PTX derivative was placed in a glass vial, followed by the addition of 10 ml of a DCM and methanol mixed solution (v:v = 9:1) to dissolve it. After filtration, the filtrate was transferred into a 20-ml glass vial, sealed with Parafilm, and perforated with several small holes. The solution was left to stand at 25 °C to allow the solvent to evaporate slowly. When crystals appeared, they were collected and analyzed for their crystal structure using single-crystal x-ray diffraction. The resulting crystal structure was submitted to the Cambridge Crystallographic Data Centre and stored under reference number CCDC2042465.

### Preparation of the 360A-constrained AS1411–PTX complex (PSaA360)

360A iodide, a trisubstituted acridine derivative, intercalates between the G-quartet planes of the AS1411 G-quadruplex through π–π stacking interactions with the terminal G-quartet. In our synthesis, the AS1411–PTX (PSaA) conjugate was reacted with 360A iodide in dimethyl sulfoxide (DMSO) at a molar ratio of approximately 1:10 (10 nmol PSaA versus 113 nmol 360A). After 30 min, the mixture was subjected to ultrafiltration (3-kDa cutoff) to remove excess free 360A and DMSO. To evaluate the loading efficacy of 360A, the reaction mixture prior to ultrafiltration was analyzed utilizing the Agilent 1290 HPLC system (YMC-Triart C18 column, 15% to 70% acetonitrile in 100 mM TEAA, pH 7.4, over 20 min at 0.5 ml·min^−1^, ambient temperature, ultraviolet [UV] 230 nm). The standard curve exhibited a highly linear relationship with 360A across substance amounts of 0.625, 1.25, 2.5, 5, and 10 nmol (*Y* = 1,398.6*X* + 106.52, *R*^2^ = 0.999). Consequently, the amount of loaded 360A calculated by subtracting excess 360A (102 nmol) from the total amount (113 nmol) was 11 nmol, corresponding to a loading ratio of approximately 1:1 (360A to PSaA).

### Cell proliferation assay

SKOV3 cells were seeded into 96-well plates at 5 × 10^3^ cells per well and incubated overnight at 37 °C. For the sequential drug treatment, cells were first exposed to the telomerase activator epitalon (300 nM) for 12 h. Then, PTX (5.5 nM) was added and maintained for the next 24 h. Finally, the telomerase inhibitor Telomerase-IN-3 (30 nM) was added and cells were incubated for the remaining 12 h. The experimental groups included vehicle, epitalon alone, Telomerase-IN-3 alone, PTX alone, epitalon plus PTX (E/P), and epitalon plus PTX plus Telomerase-IN-3 (E/P/T). After 48 h, cell viability was evaluated using the CCK-8 kit (HY-K0301), with the optical density measured at 450 nm after 1-h incubation (SpectraMax i3x).

To evaluate the inhibitory effects of PSaA360 on ovarian cancer and normal cells, SKOV3, SKOV3/PTX, A2780, A2780/PTX, and IOSE-80 cells were seeded at 5 × 10^3^ cells per well into 96-well plates and incubated overnight. The cells were then treated with vehicle, PTX, PTX360 (free PTX plus 360A), or PSaA360 for 48 h. Cell viability was determined using the same CCK-8 protocol as described above.

### FCM analysis

The Cy3 fluorescence intensity was assessed using FCM to explore cellular uptake posttreatment. The cells including HEK293T cells, ID8 cells, A2780 cells, and SKOV3 cells were seeded into 6-well plates at a density of 2 × 10^5^ per well and adhered overnight. Solutions of AS1411, etc., were added into wells at a concentration of 50 nM for 3.5 h. Following incubation, the cells were rinsed 3 times with phosphate-buffered saline (PBS) and harvested. The cellular uptakes were detected using Beckman Coulter CytoFLEX (USA).

### Surface plasmon resonance analysis

The BIAcore X100 system was used to monitor the interaction between NCL protein and AS1411, etc. GE Healthcare’s CM5 sensor chips were utilized for immobilizing NCL protein via an amine-coupling procedure using NHS and 1-ethyl-3-(3-dimethylaminopropyl)carbodiimide (EDC) reagents. A running buffer of 1× PBS at 25 °C was employed after filtering and degassing. The chip surface was washed with PBS at 10 μl·min^−1^ postloading. EDC and NHS were circulated at 5 μl·min^−1^ for 10 min to activate carboxyl groups. NCL protein was injected at 5 μl·min^−1^ for 5 min, followed by hydroxyethylammonium chloride to block carboxyl groups. The system was equilibrated with PBS for 3 h. AS1411, etc., initially at 2 μM in PBS, were diluted to various concentrations and injected over the sensor surface for 3 min at 30 μl·min^−1^ with a 5-min dissociation time. The data were analyzed using a multicycle kinetics mode with a 1:1 binding fit [36].

### CD analysis

A J-715 CD spectropolarimeter was employed to examine the secondary configurations of AS1411, etc., in 1× PBS buffer (137 mM NaCl, 2.7 mM KCl, 10 mM disodium hydrogen phosphate buffer, and 1.8 mM KH_2_PO_4_, pH 7.4). The baseline signal of the binding solution was gauged and deducted from the CD profile. Before the evaluation, the enclosure was flushed with desiccated refined nitrogen (99.99%) and maintained under a nitrogen environment during the trials. CD profiles were documented at 0.1-nm intervals spanning from 320 to 220 nm, recording at a pace of 20 nm·min^−1^ with a 1-nm width and a 4-s time constant at 25 °C [37].

### Thermal stability analysis

The thermal stability of aptamers was examined by determining the *T*_m_ values of AS1411, etc. The UV melting profiles of the aptamers were observed using an Agilent Cary 100 UV–visible spectrometer that was outfitted with a temperature controller. The absorbance of each sample (2.5 μM) in binding buffer (100 mM Hepes, pH 7.5, 150 mM NaCl) was monitored at 260 nm with the temperature increased from 25 to 95 °C at a heating rate of 1 °C·min^−1^. Each *T*_m_ value was derived from the first derivative of the melting curve utilizing the Cary WinUV software.

### Serum stability analysis

The stability of aptamers in 10% serum was assessed using polyacrylamide gel electrophoresis (PAGE) in a Bio-Rad instrument. Each sample (AS1411, etc., 100 μM) was incubated in 10% serum at 37 °C, collected at specified time intervals (ranging from 0 to 72 h), mixed with DNA urea-PAGE loading buffer, heated at 95 °C for 5 min, and then analyzed using 15% urea denaturing PAGE gel and electrophoresed at 80 V for 2.5 h in 0.5× tris–borate–ethylenediaminetetraacetic acid running buffer. The gels were stained with GenGreen nucleic acid gel stain for 20 min, and the DNA bands were visualized using a fluorescence gel imaging analyzer.

### Confocal microscopy analysis

SKOV3 cells were plated into confocal observation dishes at a density of 2 × 10^4^ cells per well and adhered overnight. Solutions containing AS1411, etc., were introduced into the wells at a concentration of 50 nM and incubated for 3.5 h. Following incubation, the cells were rinsed twice with PBS and harvested. The red fluorescence was visualized using a Nikon Eclipse Ts2 fluorescence microscope.

### 360A loading analysis

Using the updated HPLC conditions (XBridge Oligonucleotide BEH C18 column, 5% to 60% acetonitrile in 50 mM TEAA, pH 7.2, over 30 min at 1.0 ml·min^−1^, ambient temperature, UV 230 nm) and the new standard curve (*Y* = 814.3*X* − 3,853, *R*^2^ = 0.994), the loading efficiency of 360A into PSaA was evaluated at 3 molar ratios (360A:PSaA = 1:3, 1:6, and 1:9) by fixing PSaA at 10 nmol and adding 30, 60, or 90 nmol of 360A, respectively. Then, each mixture was subjected to ultrafiltration (3-kDa cutoff) to remove unbound 360A. Prior to ultrafiltration, the amount of loaded 360A was calculated by subtracting the excess free 360A (quantified via HPLC using the standard curve) from the total added. The resulting loading ratios (loaded 360A/PSaA) for the 1:3, 1:6, and 1:9 inputs were thereby determined as 0.47:1, 1.08:1, and 1.14:1, allowing comparison with the previously observed ~1:1 loading at a 1:10 input ratio.

### Transwell analysis

The migratory and invasive abilities of SKOV3 cells were assessed by transwell inserts for migration assay and Matrigel-coated invasion chambers for invasion assay, respectively. SKOV3 cells were seeded at 3.5 × 10^4^ per well, into the top chambers (for migration or invasion assays), and complete medium was added to the lower chamber as chemoattractant. Then, the cells were treated with vehicle, PTX, PTX360, and PSaA360 at 5.5 nM and allowed to migrate/invade for 24 h. Subsequently, the cells were rinsed with PBS, fixed in 4% PFA, and stained with crystal violet for 15 min. The nonmigrated/invaded cells were removed from the upper side of the membrane using cotton swabs. The cells that had migrated/invaded to the basal side of the membrane were visualized using a Leica DMI3000 microscope (Germany) at 40× magnification. The migrated/invaded cells in 5 randomly chosen fields were quantified using the ImageJ software.

### Wound-healing analysis

SKOV3 cells were seeded into each well of a Culture-Insert (ibidi) placed into a 6-well culture plate at an absolute cell number of approximately 20,000 per insert well. After overnight adhesion at 37 °C to form a confluent monolayer, the insert was gently removed to create a defined cell-free gap. The epithelial ovarian cancer cells were then treated with PTX, PTX360, and PSaA360 for 24 h at 37 °C. Images of the wound area were captured at 0 and 24 h using a fluorescence microscope (Nikon Eclipse Ts2, Japan) for observation and analysis.

### Colony formation analysis

SKOV3 cells were seeded into 6-well plates with complete DMEM at a density of 1 × 10^3^ cells per well and adhered overnight. Then, the cells were treated with vehicle, PTX, PTX360, and PSaA360 at 1.375 nM, and the medium was replaced every 2 d until visible colonies were formed. After incubation for 7 to 14 d, cells were rinsed with PBS, fixed in 4% PFA, and subsequently stained with crystal violet for 15 min. Then, the crystal violet was removed with tap water. Images were photographed using ImageScanner III (USA) under reflective settings.

### Live-cell staining analysis

SKOV3 cells were seeded into 6-well plates with complete DMEM at a density of 5 × 10^4^ cells per well and adhered overnight. Then, the cells were treated with vehicle, PTX, PTX360, and PSaA360 at 5.5 nM. After 24-h incubation, the cells were washed with PBS and stained with staining solution (5 μl of 4 mM calcein AM in 10 ml of PBS). After incubation at room temperature in the dark for 20 min, the images were visualized using a Leica DMI3000 microscope (Germany).

### Cell adhesion analysis

The 24-well plates were coated with Matrigel/serum-free minimum essential medium (1:300 ratio) and air-dried overnight in a biosafety cabinet. Then, the plate was washed with PBS to remove excess gel. Meanwhile, SKOV3 cells were seeded into 6-well plates with complete DMEM at a density of 5 × 10^4^ cells per well and adhered overnight. Then, the cells were treated with vehicle, PTX, PTX360, and PSaA360 at 5.5 nM. After 48-h incubation, the cells were collected, washed, and seeded into a Matrigel-coated 24-well plate at a density of 4 × 10^3^ cells per well. After 20-min incubation, the cells were washed with PBS, fixed with methanol for 15 min, and stained with Giemsa for 15 min. The images were visualized using a Leica DMI3000 microscope (Germany).

### Animal study for antitumor efficacy

The animal facility ensured a controlled environment with regulated temperature and a consistent 12-h light/dark cycle, providing mice with ad libitum access to food and water throughout the study. A minimum acclimation period of 1 week was allotted for the mice before any experiments were initiated.

Female BALB/c nude mice, 8 weeks old, were subcutaneously inoculated with 2 × 10^6^ SKOV3 cells in the armpit. Upon tumor formation within 3 weeks, the mice were randomly assigned to 4 groups for further investigations. The mice received intravenously vehicle, PTX, PTX360, and PSaA360 twice a week for 4 weeks at a dosage of 5.85 μmol·kg^−1^ (equivalent to PTX 5 mg·kg^−1^), with the vehicle group receiving an equivolume of PBS. Tumor dimensions and body weight were recorded biweekly with intervals of 3 to 4 d. Tumor size and body weight were assessed twice weekly, with 3- to 4-d intervals between measurements. Upon completion of the treatment, the mice were humanely euthanized, and tumor weights were recorded.

### Distribution effect in vivo

Female BALB/c nude mice, 8 weeks old, were subcutaneously inoculated with 2 × 10^6^ SKOV3 cells in the armpit. Upon tumor formation within 3 weeks, the mice were randomly assigned to 3 groups for further experimentation. The mice received intravenously vehicle, Cy3-labeled PSaA, and Cy3-labeled PSaA360 at a dosage of 5.85 μmol·kg^−1^ (equivalent to PTX 5 mg·kg^−1^), with the vehicle group receiving an equivolume of PBS. After 2-h treatment, the mice were euthanized. Two mice were administered with PBS as a control. The Cy3 fluorescence intensities in major viscera (heart, liver, spleen, lung, and kidney) and tumor at the organ level were detected.

### Histological examination

Paraffin slices underwent a sequence of immersion stages, commencing with eco-friendly dewaxing transparent liquids I and II, anhydrous ethanol I and II, and 75% ethyl alcohol, followed by washing with tap water. Cryogenic slices were defrosted and fixed using a tissue fixation solution and then washed with flowing water. For hematoxylin staining, the slices were submerged in hematoxylin solution and treated with hematoxylin differentiation solution and hematoxylin bluing solution, with washes interspersed. Eosin staining entailed immersing slices in 85% ethanol, 95% ethanol, and eosin dye. Dehydration and sealing phases involved various ethanol and xylene solutions, culminating in sealing with neutral gum. Following these processes, microscopic examination, image capture, and analysis were conducted. The results exhibited nuclei in blue and cytoplasm in pink upon interpretation.

### IHC analysis

The paraffin slices underwent the dewaxing process by being immersed in eco-friendly dewaxing solutions I to III and anhydrous ethanol I to III, followed by washing with purified water. Specific antigen retrieval was carried out using a buffer, and the slides were washed with PBS on a rocking platform. Next, the natural peroxidase activity was neutralized by treating the slides with a 3% hydrogen peroxide solution and rinsing them with PBS. Subsequently, the slices were blocked with 3% bovine serum albumin to reduce nonspecific binding. The primary antibody Ki-67 (GB111499, 1:1,000) and the secondary antibody (GB23303, 1:200) were applied sequentially, with appropriate washing steps in between. 3,3′-Diaminobenzidine staining was utilized to visualize the target antigen, and counterstaining with hematoxylin was performed to highlight cellular structures.

The sections were dehydrated using a sequence of alcohol and xylene solutions before being mounted. The prepared slides were then scrutinized under a bright-field microscope for result interpretation.

### Fluorescence TUNEL analysis

TUNEL staining for cell apoptosis was performed in tumor tissues. First, the sections were dewaxed with environmentally friendly solutions (Servicebio, G1128) and ethanol (Sinopharm Group Chemical Reagent Co., LTD, 100092183), followed by protease K repair. The sections were balanced at room temperature, and a reaction solution containing terminal deoxynucleotidyl transferase enzyme was applied to cover the tissue. After incubation, 4′,6-diamidino-2-phenylindole (DAPI) staining of nuclei was performed. The sections were then sealed, and microscopic photography was conducted. In the interpretation of the results, nuclei stained with DAPI appeared blue under UV light, while apoptotic nuclei labeled with tetramethylrhodamine fluorescein from the TUNEL kit (Servicebio, G1502) emitted red fluorescence.

### Transcriptomics analysis

Total RNA was extracted from samples, and its quality was assessed using a Qubit 4.0 fluorometer and Qsep400 bioanalyzer. Messenger RNA (mRNA) was enriched via oligo(dT) magnetic beads, fragmented, and reverse-transcribed into complementary DNA (cDNA). After end repair, A-tailing, adapter ligation, and PCR amplification, libraries were sequenced on an Illumina platform. Raw reads were quality-filtered using fastp (v0.23.2), and clean reads were aligned to the human reference genome (GRCh38, Ensembl v109) with HISAT2 (v2.2.1). Gene expression was quantified as fragments per kilobase of transcript per million mapped reads (FPKM) using featureCounts (v2.0.3). Differential expression analysis (|log_2_FC| ≥ 1, false discovery rate < 0.05) was performed with DESeq2 (v1.38.3). Gene set enrichment analyses were conducted using clusterProfiler (v4.6.0). The raw sequencing data have been deposited in the NCBI SRA under accession number PRJNA1440175.

### qPCR analysis

SKOV3 cells were seeded at a density of 1.0 × 10^6^ cells per well and incubated overnight for adherence. PSaA360 was prepared in medium at the specified concentration, added to the wells, and incubated for 48 h at 37 °C. Total RNA was extracted using 1 ml of TransZol Up reagent and 0.2 ml of chloroform according to the manufacturer’s protocol (TransZol Up Plus RNA Kit). The RNA was further purified using mRNA spin columns, and its concentration was measured. For each sample, 1.0 μg of total RNA was reverse-transcribed into cDNA using a high-capacity cDNA reverse transcription kit on T100 Thermal Cycler (Bio-Rad). qPCR reactions were performed using SYBR Green qPCR Mix on 7900 HT Sequence Detection System (Applied Biosystems, USA). The relative expression levels of *TNFSF15*, *PDX1*, *CLEC4M*, *CNR2*, *CDKN1A*, *BAD*, *CD24*, *HTR2C*, *AQP4*, *SLC25A2*, *NRK*, *IFIT1B*, and *RFX6* were determined using the 2^−ΔΔCt^ method [38], with *GAPDH* as the endogenous normalizer. All reactions were performed in triplicate. Statistical significance was calculated using one-way analysis of variance (ANOVA) with Tukey’s post hoc test, and *P* < 0.05 was considered significant. The primer sequences are listed in Table S3.

### Preparation of the AS1411 structure

The structural groundwork involved the utilization of Z-G4 containing d[T(GGT)4TG(TGG)3TGTT] (Protein Data Bank [PDB] ID: 4U5M) as a reference due to its resemblance to AS1411 [39]. AS1411 was adapted based on this template, with adjustments made for its initial form. Specifically, the penultimate thymine (T) was switched to guanine (G), and the terminal thymine bases were removed via Discovery Studio. The preliminary structure of AS1411 was obtained, and simultaneously, the coordinates for human NCL RBD1,2 (PDB ID: 2KRR) were retrieved from the Research Collaboratory for Structural Bioinformatics, https://www.rcsb.org/). This dataset comprised 20 refined structures, exhibiting notable discrepancies across various conformations. Among all modeled structures, model 9 best matched the known key interaction regions, with the AS1411 5′ cap stacking on RBD1 and the central loop positioned near RBD2’s β-sheet [40]. Therefore, the ninth model was selected to serve as the foundational structure for human NCL RBD1 and RBD2.

### Protein–aptamer molecular docking

A semiflexible docking approach was employed to conduct the molecular docking of the aptamer and the small molecule. In essence, this technique maintains the protein receptor in a rigid conformation while allowing the ligand to explore diverse conformations during the semiflexible docking phase. The semiflexible docking algorithm of AutoDock 4.2.6 was utilized as the default setting for this process. Simultaneously, the HDOCK server (http://huanglab.phys.hust.edu.cn/software/hdocklite/) was employed to predict the binding modes of proteins and aptamers [41]. Rigid-body docking was performed for each pair of receptor and ligand, generating the top 10 predicted docking outcomes for visualization purposes. The model exhibiting the highest docking score was chosen as the initial structure for the protein–aptamer complex, which was subjected to subsequent MD simulations.

### MD analysis

The complexes formed by the aptamer and NCL RBD1,2 underwent MD simulations using GROMACS 2023. These simulations employed the Amber99SB force field. Each complex was positioned inside a cubic box containing water molecules, with a minimum distance of 8 Å maintained between the solute and the box boundaries. To maintain system neutrality, potassium or chloride ions were introduced to reach a concentration of 150 mM, a level confirmed to support the proper folding of AS1411. The system underwent an initial minimization step comprising 1,000 iterations. Subsequently, the system’s temperature gradually rose from 0 to 300 K over 100 ps, with a pre-equilibration phase. A production run lasting 20 ns was then conducted. Following the completion of the MD simulations, trajectory analysis was performed using GROMACS 2023 and gmx_MMPBSA. The conformation with the lowest total energy over time was chosen based on the system’s energy profile. Salt bridge analysis was conducted using the PLIP online tool [42] and visualized using PyMOL (version 2.3.0). The simulation results were analyzed to assess RMSF [43], RMSD, total energy, and binding free energy.

### Statistical analysis

The data are reported as mean ± SD. For comparisons among multiple groups, a one-way ANOVA with Tukey’s post hoc test was employed. For comparisons of the interaction between 2 independent variables, a 2-way ANOVA with Sidak’s post hoc test was employed [44]. For comparisons between 2 independent groups, an unpaired 2-tailed Student *t* test was applied when both normality and variance homogeneity assumptions were met; otherwise, the nonparametric Mann–Whitney *U* test was used. Statistical analyses were performed using GraphPad Prism 8, with statistical significance defined as *P* < 0.05.

## Ethical Approval

All animal experiments were approved by the Animal Experimentation Ethics Committee of Hong Kong Baptist University and conducted in accordance with relevant guidelines (approval no.: REC/24-25/0407). Human placental tissues were obtained from donors at the Clinical Ethics Management Committee of Nan Fang Hospital of Southern Medical University. All procedures involving human samples were approved by the Ethics Committee (approval no.: NFEC-2019-180).

## Data Availability

The data that support the findings of this study are available in the paper and the Supplementary Materials. The raw multi-omics datasets supporting the conclusions of this study can be obtained from the corresponding authors upon reasonable request.
