# Supplementary material for: Dual-Mechanism Aptamer–Drug Complex Overcomes Paclitaxel Resistance in Ovarian Cancer via Structural Constraint and Telomerase Inhibition
Source: Research (Wash D C). 2026 Jul 14;9:1362. doi: 10.34133/research.1362 (PMC13365583; doi:10.34133/research.1362)
Supplement: Supplementary 1 — Figs. S1 to S21 Tables S1 to S3 [file research.1362.f1.pdf]

Supplementary Material

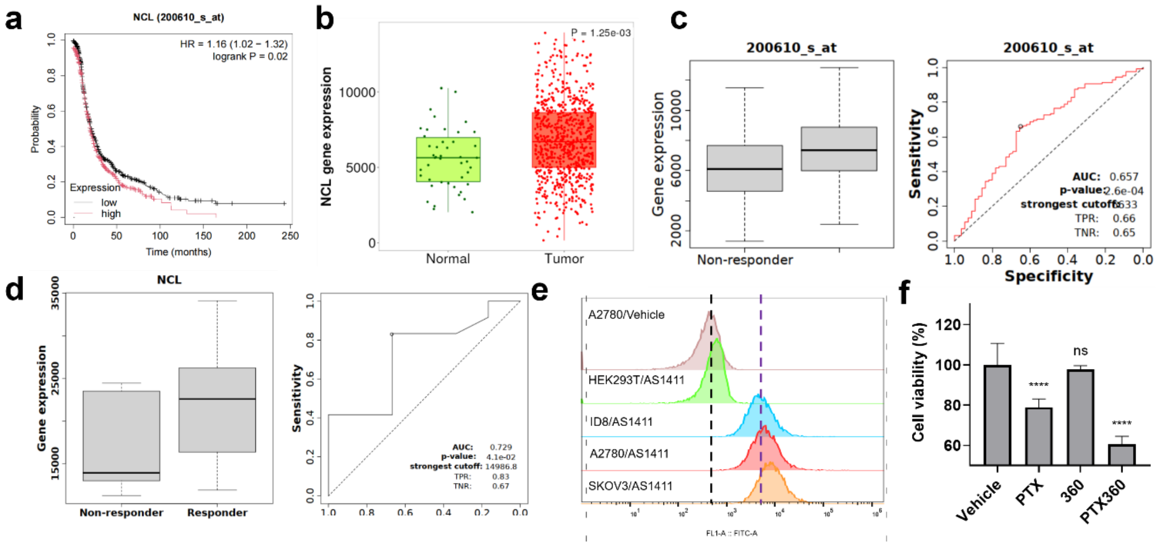

Fig. S1 Nucleolin is overexpressed in ovarian cancer and correlates with PTX response. (**a**) Kaplan-Meier analysis of progression-free survival (PFS) stratified by high versus low nucleolin (NCL) gene expression. (**b**) NCL gene expression in normal versus ovarian tumor tissues. (**c**) NCL gene expression in PTX non-responders versus responders (left) and ROC curve for the predictive value of NCL (right) in patient samples. (**d**) NCL gene expression in PTX non-responders versus responders (left) and ROC analysis (right) in cell line models. (**e**) AS1411 (Cy3-labeled) uptake in non-cancerous (HEK293T) and ovarian cancer cells (ID8, A2780, SKOV3), detected by flow cytometry analysis. (**f**) The inhibitory effects of Vehicle, PTX, 360 and PTX360 groups on SKOV3 cells (5.5 nM). Data are presented as mean ± SD (n=6). ^{ns} P>0.05, ****P<0.0001 versus Vehicle group using one-way ANOVA with Tukey's post-hoc test.

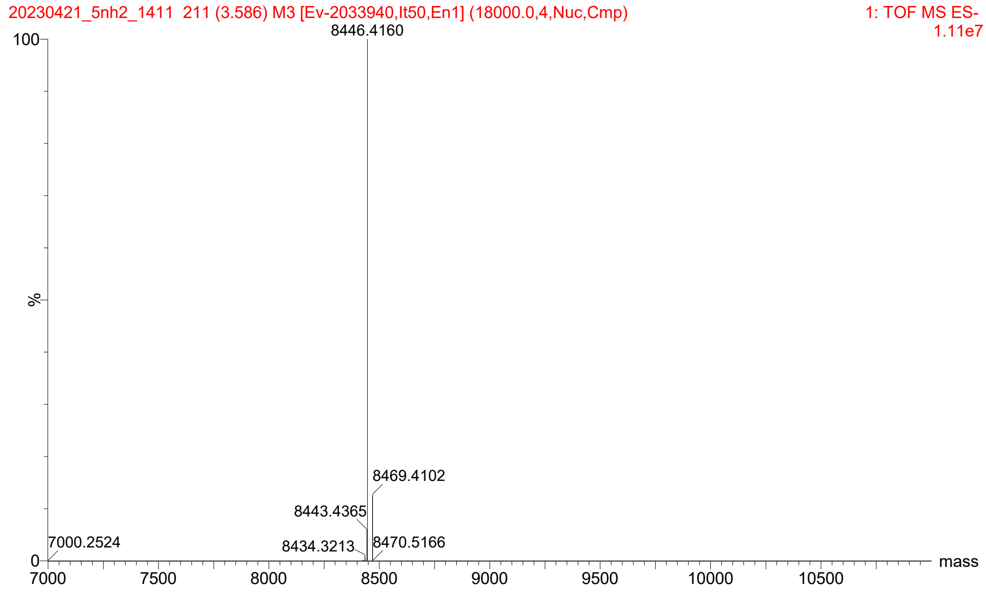

Fig. S2 ESI mass spectrum of NH_2-AS1411 (m/z, $[M + H]^{+}$): 8447.4 (calculated), 8446.4

(observed). Notes: NH_2-AS1411, 5'-amino-AS1411; ESI, electrospray ionization.

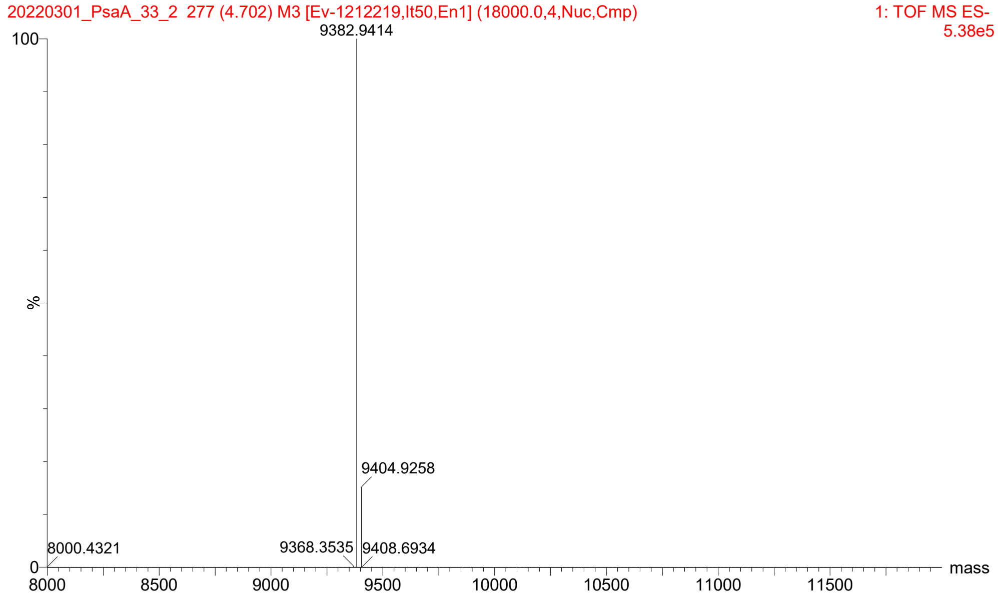

Fig. S3 ESI mass spectrum of PSaA (m/z, $[M + H]^+$): 9382.8 (calculated), 9382.9 (observed). Notes: PSaA, AS1411-paclitaxel conjugate; ESI, electrospray ionization.

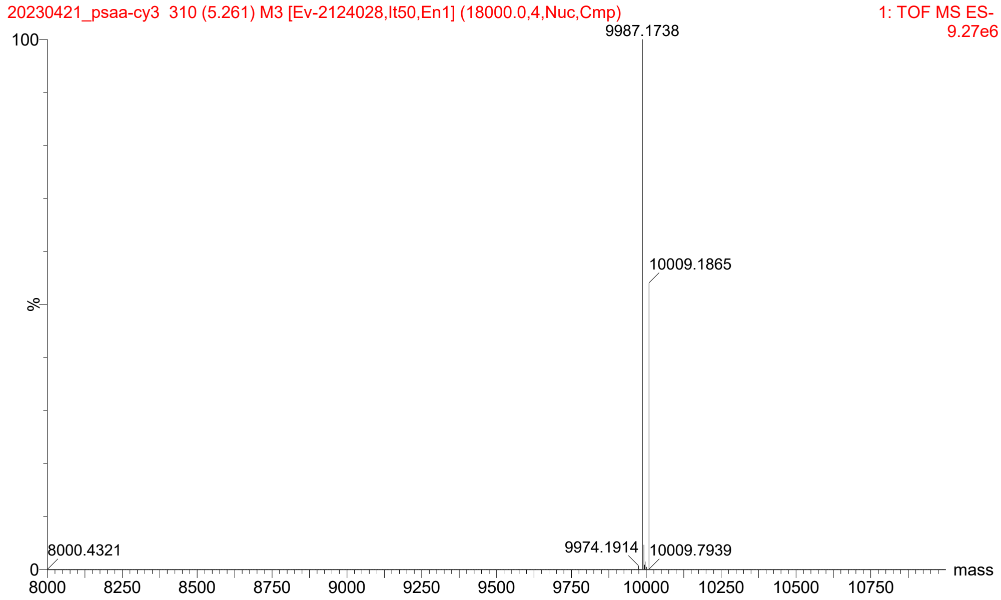

Fig. S4 ESI mass spectrum of PSaA-Cy3 (m/z, $[M + H]^+$): 9989.1 (calculated), 9987.2 (observed). Notes: PSaA-Cy3, Cy3 labeled AS1411-paclitaxel conjugate; ESI, electrospray ionization.

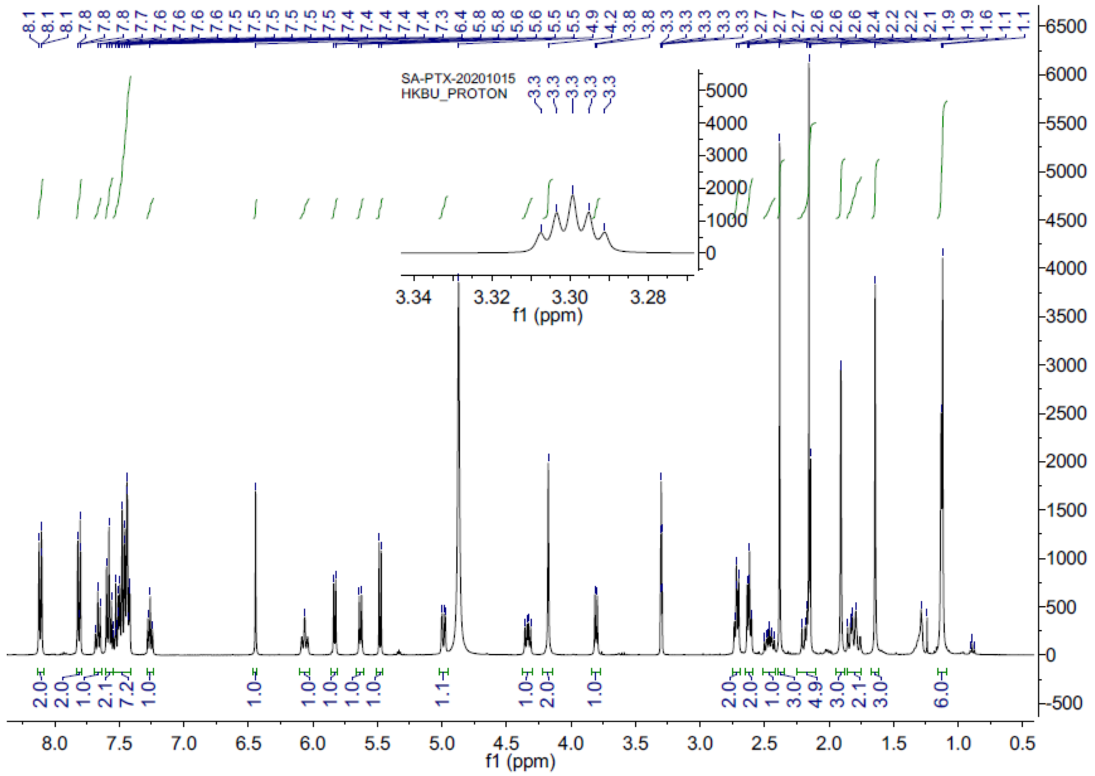

Fig. S5 ^{1}H-NMR spectrum of compound 2 in MeOD (400 MHz). Notes: ^{1}H-NMR, proton nuclear magnetic resonance; MeOD, deuterated methanol.

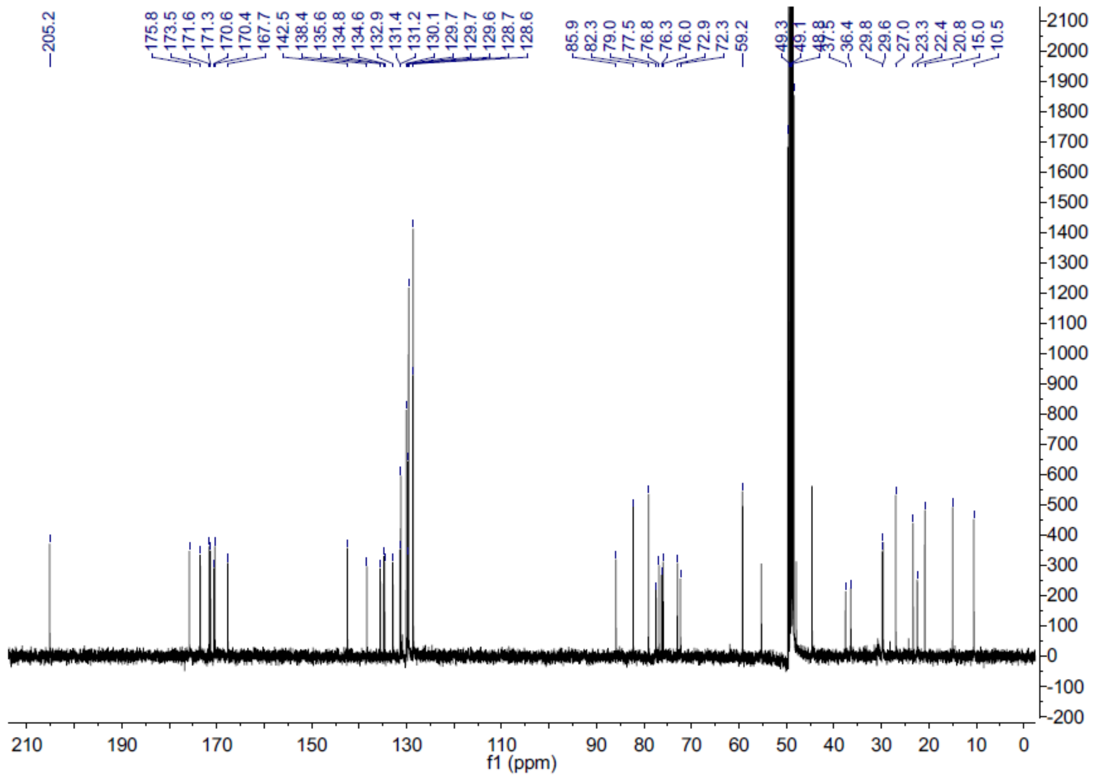

Fig. S6 ^{13}C-NMR spectrum of compound 2 in MeOD (400 MHz). Notes: ^{13}C-NMR, carbon-13 nuclear magnetic resonance; MeOD, deuterated methanol.

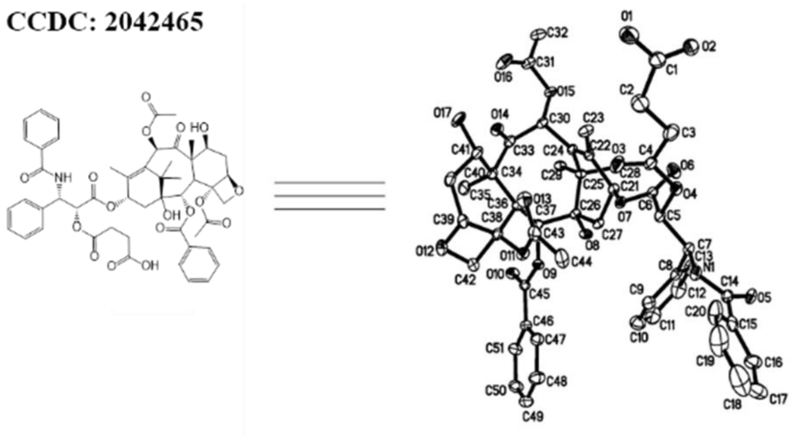

Fig. S7 X-ray crystal structure of compound 2 (100 K).

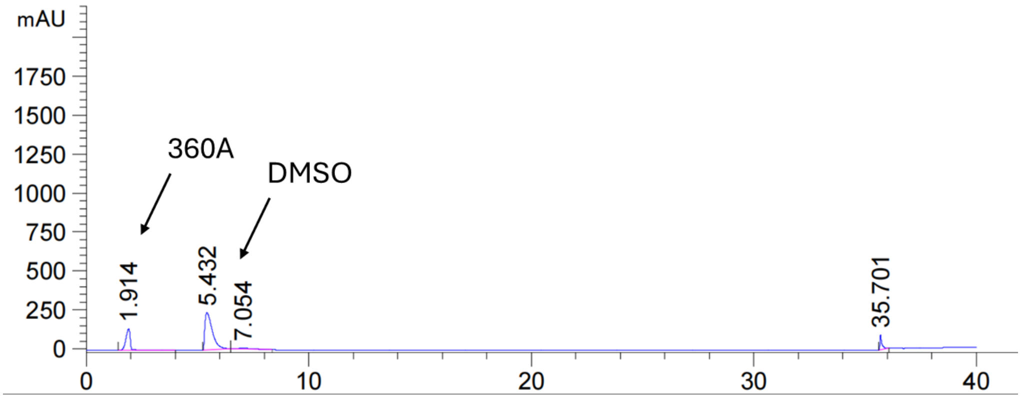

Fig. S8 HPLC spectrum at 230 nm of 360A in DMSO. The amount of 360A was 5 nmol. Notes: DMSO, dimethyl sulfoxide; HPLC, high performance liquid chromatography.

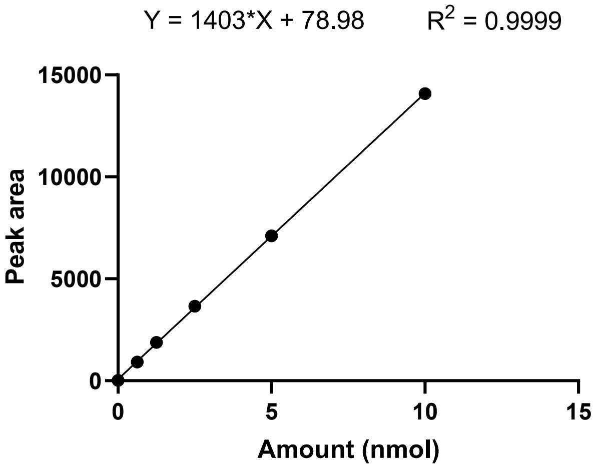

Fig. S9 Standard curve at 230 nm of the 360A.

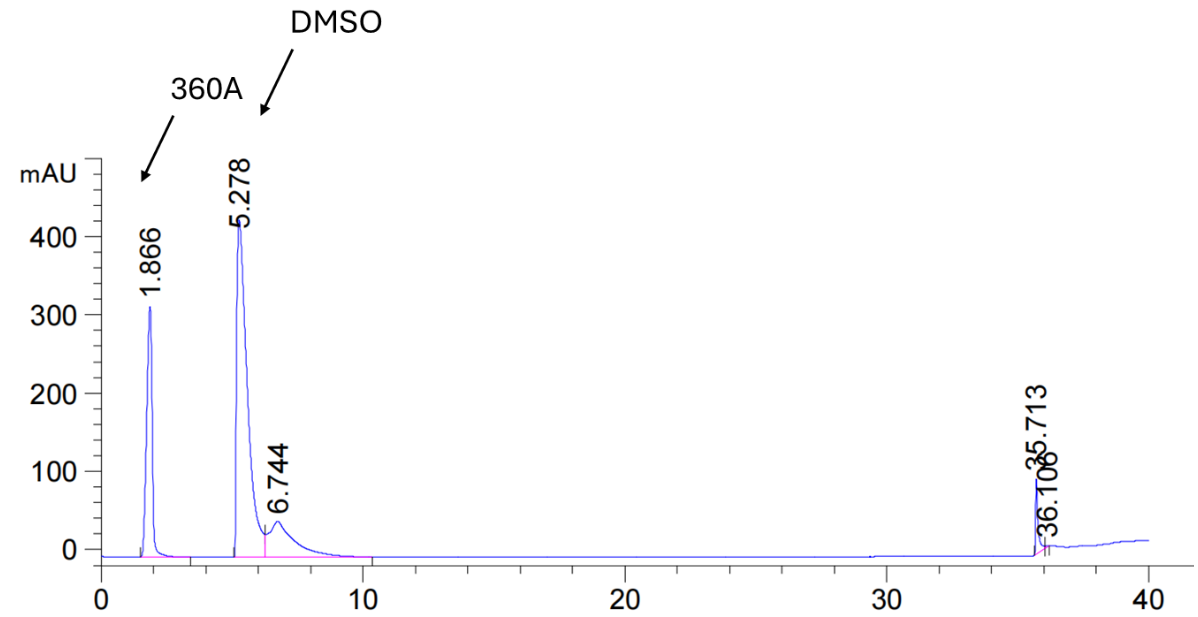

Fig. S10 HPLC spectrum at 230 nm of PSaA360 reaction mixture. The total amounts of PSaA and 360A in the mixture were 10 nmol and 113 nmol, respectively.

Quality Control | Report | Residuals | Parameters

Curve	ka (1/Ms)	kd (1/s)	KD (M)	Rmax (RU)	Conc (M)	tc	Flow (ul/min)	kt (RU/Ms)	RI (RU)	Chi² (RU²)	U-value
	1.186E+4	1.034E-4	8.721E-9	79.80		4.219E+22				43.9	33
Cycle: 4 5000 nM					5.000E-6		30.00	1.311E+23	57.66		
Cycle: 5 2500 nM					2.500E-6		30.00	1.311E+23	30.61		
Cycle: 6 1250 nM					1.250E-6		30.00	1.311E+23	20.69		
Cycle: 7 625 nM					6.250E-7		30.00	1.311E+23	13.54		
Cycle: 8 312.5 nM					3.125E-7		30.00	1.311E+23	8.974		
Cycle: 9 156.25 nM					1.563E-7		30.00	1.311E+23	3.512		

Fig. S11 The representative original images of the binding affinity of A360 to NCL, detected by the SPR assay.

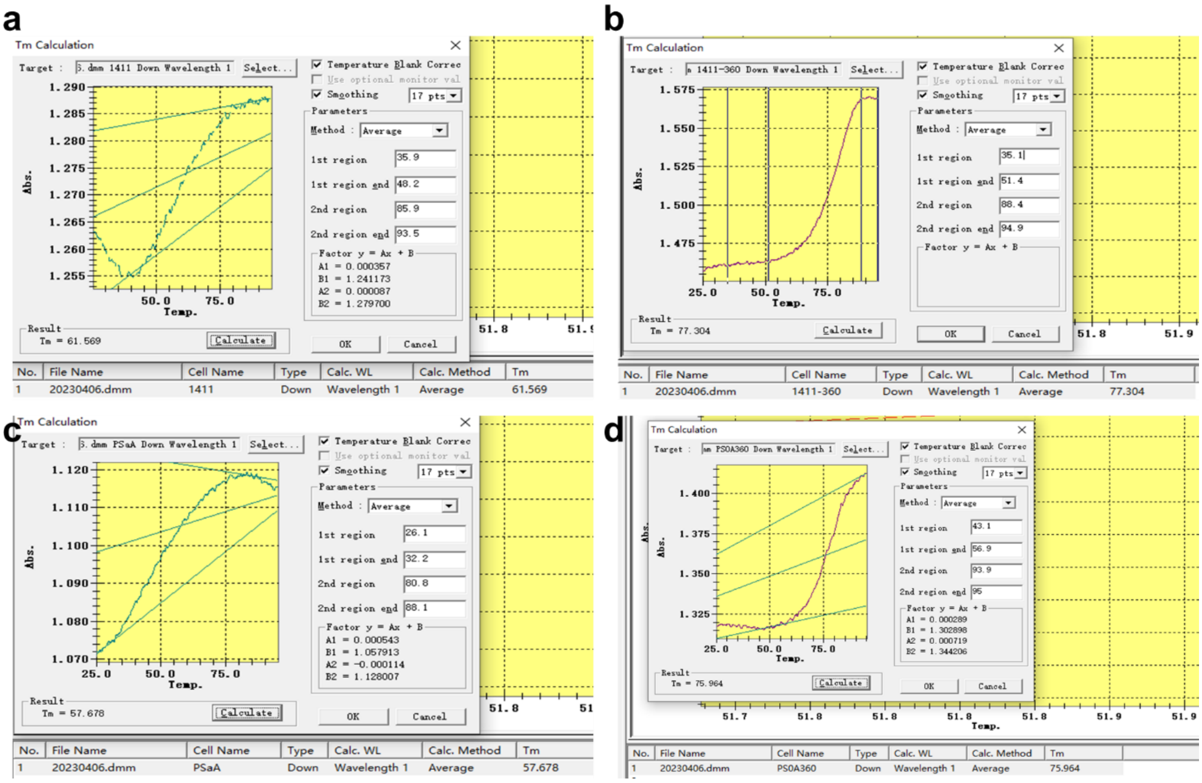

Fig. S12 The representative original images of thermodynamic stability of the (**a**) AS1411, (**b**) A360, (**c**) PSaA, and (**d**) PSaA360 groups, detected by ultraviolet spectrograph.

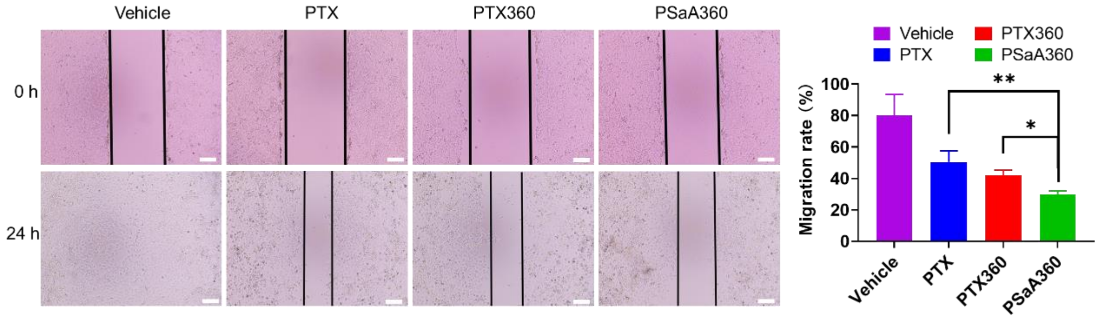

Fig. S13 The representative images and migration rate of Vehicle, PTX, PTX360, PSaA360 groups on SKOV3 cells, detected by wound healing assay. *P<0.05, **P<0.01 versus PSaA360 group using one-way ANOVA with Tukey's post-hoc test. Notes: Vehicle, PBS; PTX, paclitaxel; PSaA360, A360-paclitaxel conjugate; PTX360, paclitaxel plus 360A iodide.

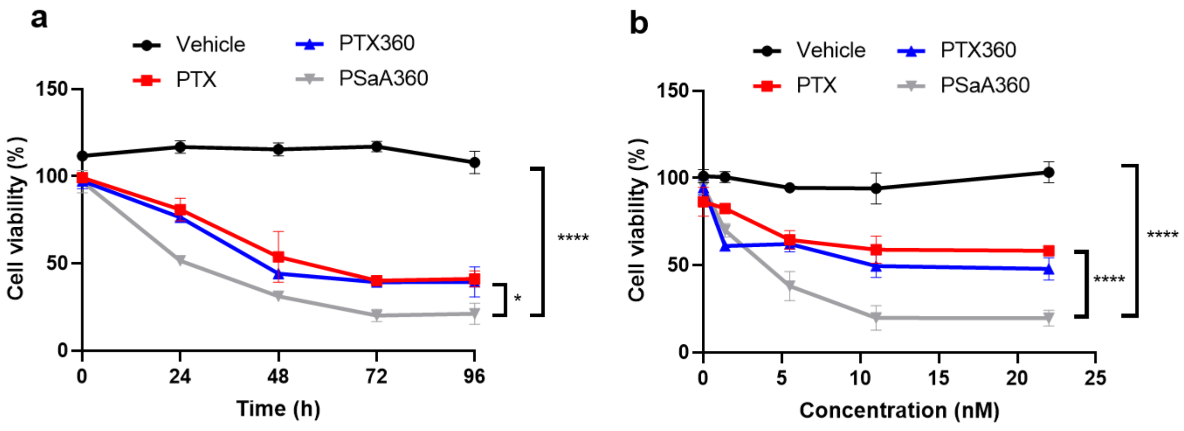

Fig. S14 The (**a**) time- and (**b**) dosage-dependent effects of Vehicle, PTX, PTX360, and PSaA360 groups on inhibiting SKOV3 cells. Data are presented as mean ± SD (n=3). Statistical significance calculated using two-way ANOVA with Sidak's post-hoc test.

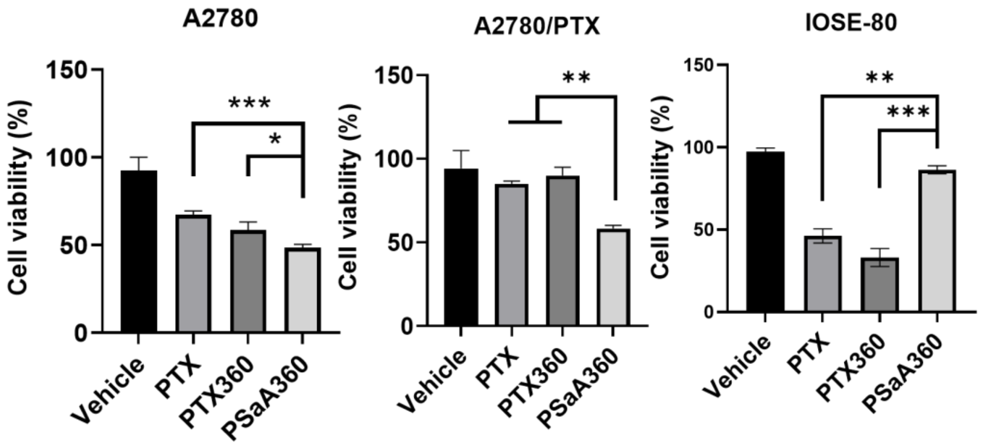

Fig. S15 The inhibitory effects of Vehicle, PTX, PTX360, and PSaA360 groups on A2780 cells (5.5 nM), A2780/PTX cells (22 nM) and IOSE-80 cells (5.5 nM). Data are presented as mean ± SD (n=3). *P<0.05, **P<0.01, ***P<0.001 versus PSaA360 group using one-way ANOVA with Tukey's post-hoc test.

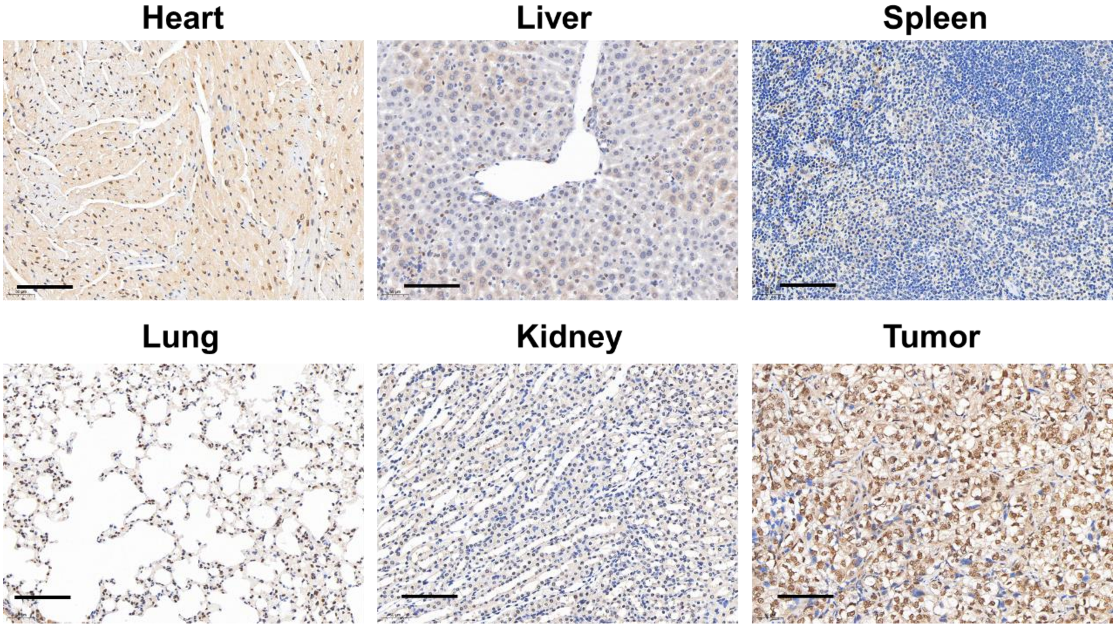

Fig. S16 The nucleolin expression distribution in the vital organs and xenografted ovarian tumors *in vivo.* NCL immunohistochemical staining analysis of heart, liver, spleen, lung, kidney and tumor sections from SKOV3-inoculated BALB/c nude female mice. NCL-positive staining was indicated by brown, and nuclei were stained by blue. Scale bars, 100 μm. Notes: NCL, nucleolin.

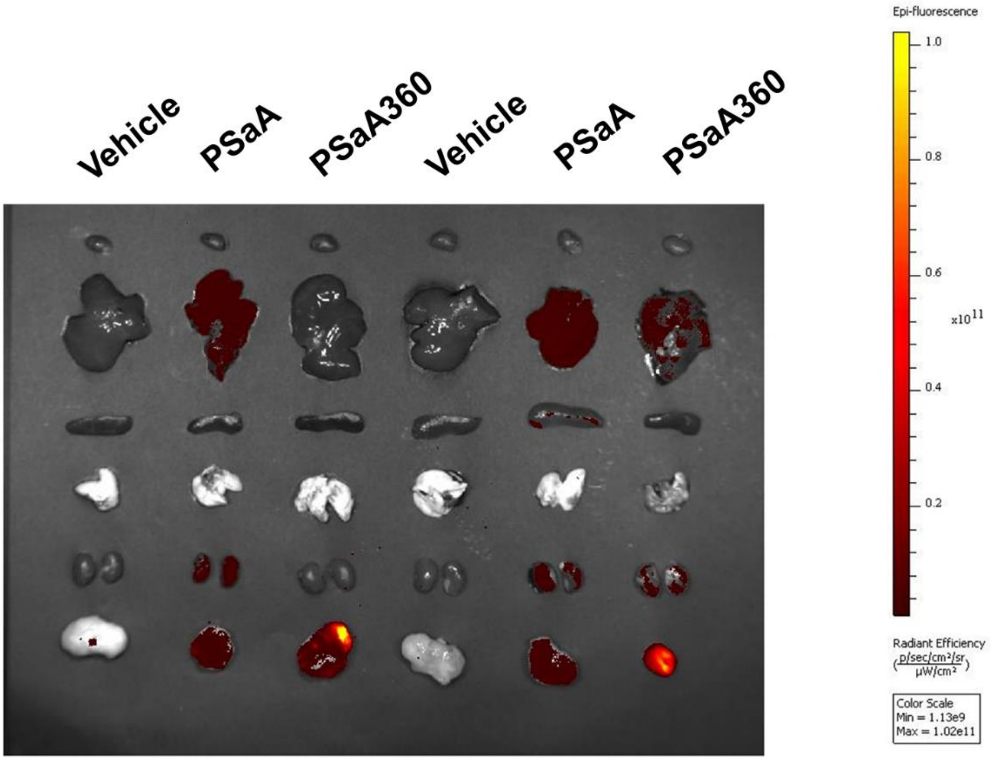

Fig. S17 PSaA360 exhibited high tumor accumulation in SKOV3 inoculated mice. Fluorescent views of the PSaA360 in major organs (heart, liver, spleen, lung, and kidney) and tumor at organ level 2 h after intravenous injection of Vehicle, PSaA and PSaA360 for drug

distribution.

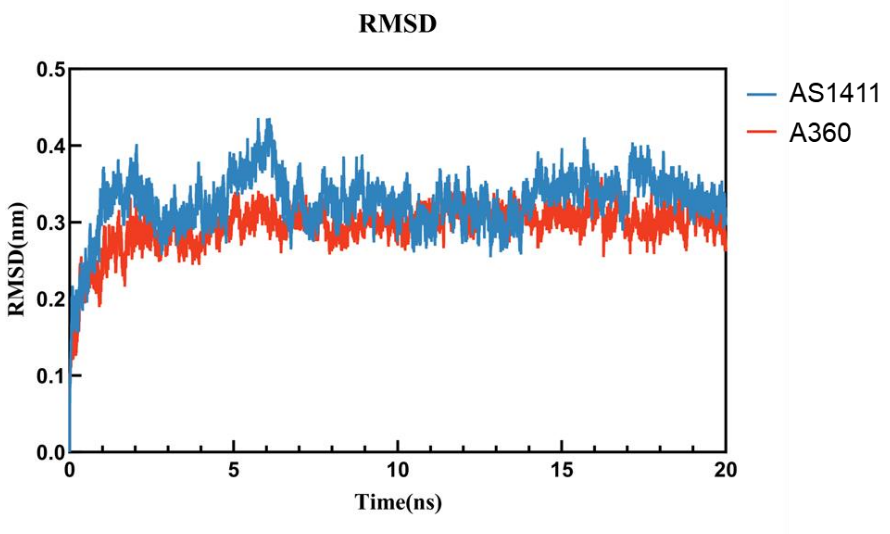

Fig. S18 The RMSD curves of AS1411 and A360 evaluated by molecular dynamics simulation. Notes: RMSD, Root mean square deviation; AS1411, nucleolin aptamer; A360, 360A iodide-constrained AS1411.

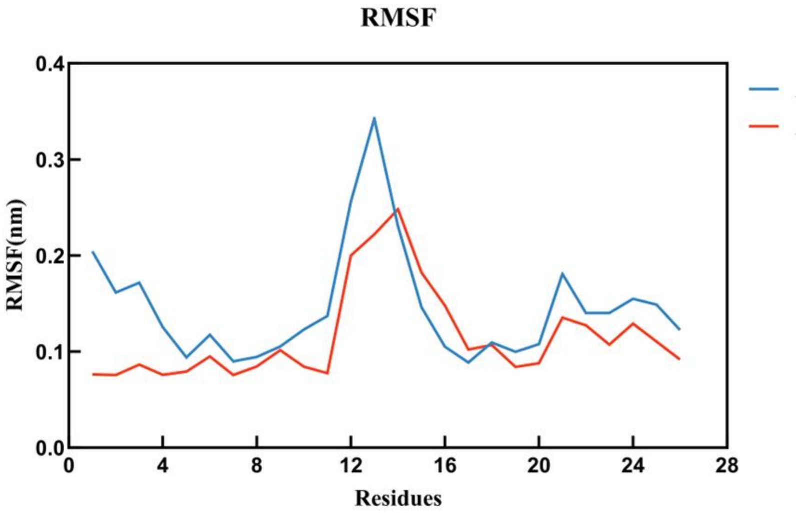

Fig. S19 The RMSF curves of AS1411 and A360 evaluated by molecular dynamics simulation. Notes: RMSF, Root mean square fluctuation; AS1411, nucleolin aptamer; A360, 360A iodide-constrained AS1411.

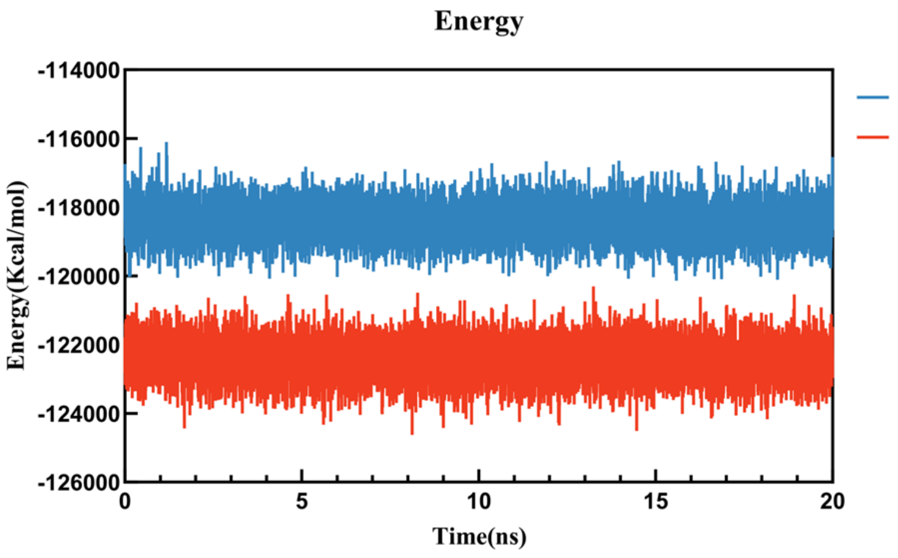

Fig. S20 The binding energy curves of AS1411 and A360 evaluated by molecular dynamics simulation. Notes: AS1411, nucleolin aptamer; A360, 360A iodide-constrained AS1411.

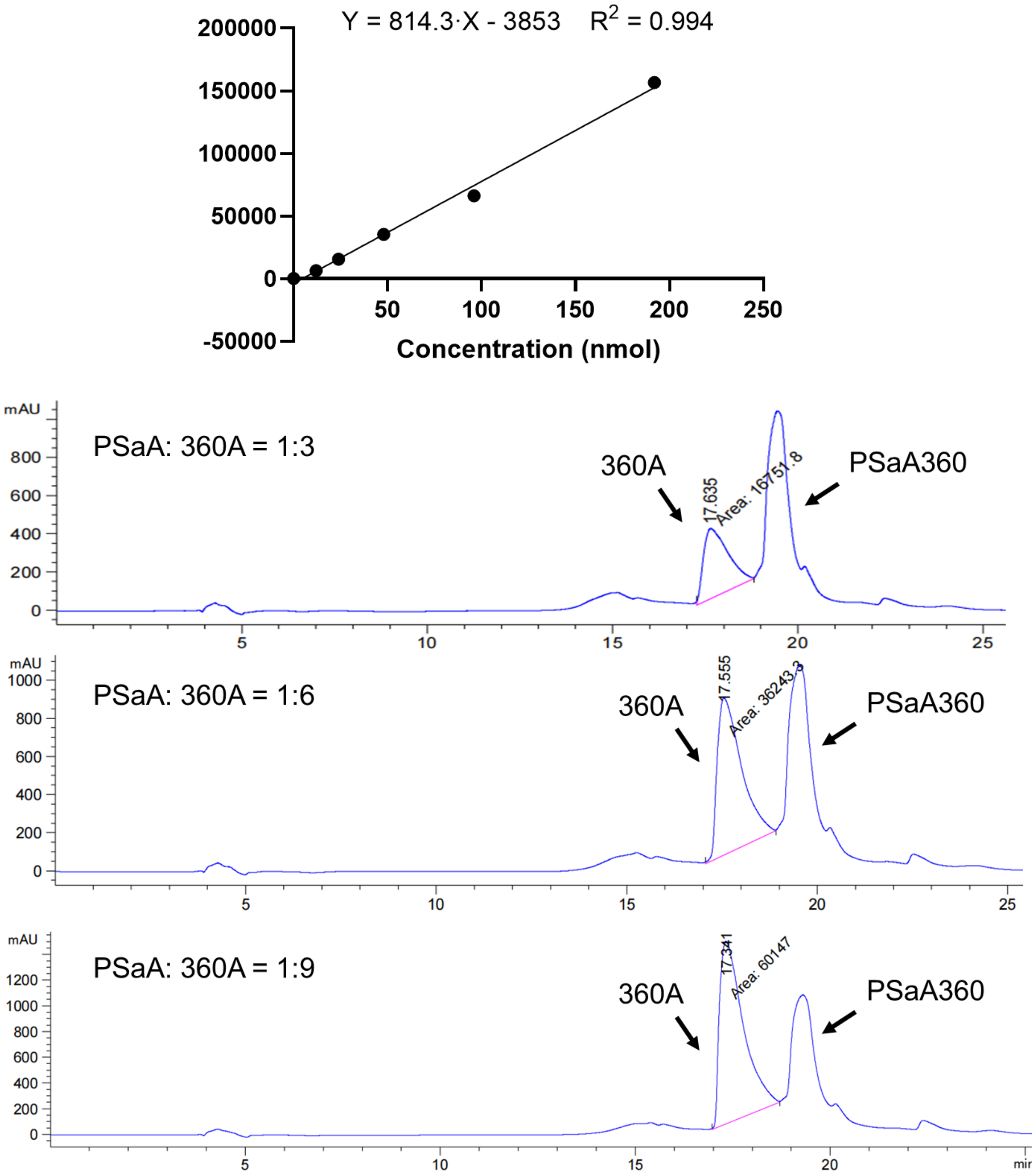

Fig. S21 The preparation of PSaA360 at different ratios.

Table S1. The original data of CD assay

Wavelength	AS1411	PSaA	A253	A360	PSaA253	PSaA360
230	-0.10	-1.59	0.34	2.41	1.96	2.52
231	-0.95	-1.03	-0.06	0.13	0.68	1.36
232	-1.67	-0.71	-0.41	-1.85	-0.36	0.31
233	-2.27	-0.62	-0.73	-3.53	-1.17	-0.62
234	-2.76	-0.70	-1.00	-4.92	-1.78	-1.44
235	-3.14	-0.93	-1.23	-6.04	-2.20	-2.14
236	-3.41	-1.28	-1.40	-6.88	-2.45	-2.71
237	-3.59	-1.72	-1.53	-7.47	-2.55	-3.15
238	-3.68	-2.21	-1.60	-7.81	-2.52	-3.45

239	-3.69	-2.73	-1.61	-7.91	-2.38	-3.62
240	-3.62	-3.23	-1.56	-7.78	-2.15	-3.64
241	-3.48	-3.69	-1.44	-7.43	-1.84	-3.52
242	-3.28	-4.08	-1.26	-6.87	-1.48	-3.24
243	-3.40	-4.09	-0.90	-6.42	-1.56	-3.36
244	-3.04	-3.96	-0.67	-5.42	-1.41	-2.71
245	-2.53	-3.58	0.12	-3.81	-1.14	-1.39
246	-2.47	-3.04	0.86	-2.28	-0.87	-0.25
247	-2.15	-2.46	1.30	-0.76	-0.37	1.08
248	-1.38	-1.58	2.10	0.82	-0.02	2.41
249	-0.12	-0.82	2.79	2.13	0.27	4.31
250	0.76	0.38	3.36	4.21	1.00	5.94
251	2.20	1.97	3.86	6.49	1.91	7.86
252	3.47	3.23	4.13	8.22	2.19	10.19
253	4.49	4.32	4.73	10.21	3.22	12.40
254	5.89	5.66	5.76	12.29	4.32	14.86
255	7.13	6.57	6.00	13.75	4.77	16.93
256	8.17	7.88	6.74	15.54	5.75	19.16
257	9.67	9.02	7.11	17.83	6.70	21.36
258	11.19	9.93	7.27	19.87	7.72	23.30
259	12.65	10.42	7.31	21.55	8.45	24.70
260	13.43	11.15	7.35	23.25	9.10	26.18
261	14.57	11.67	7.44	24.59	9.95	27.56
262	15.47	12.05	7.66	25.75	10.57	28.20
263	15.66	12.59	7.54	26.72	11.38	28.60
264	16.04	12.22	7.63	26.70	11.78	28.91
265	15.58	12.01	7.20	26.92	12.06	28.83
266	15.44	12.19	7.10	26.56	11.91	28.29
267	14.88	12.24	7.00	26.25	11.52	27.61
268	14.85	12.01	6.30	25.18	11.10	27.01
269	14.03	11.47	6.00	24.14	10.43	25.68
270	13.13	10.98	5.62	22.99	9.48	24.16
271	12.71	10.45	5.01	21.88	9.20	22.45
272	12.42	10.08	4.45	20.05	8.29	20.86
273	11.94	9.29	3.74	18.28	7.52	19.32
274	11.01	8.88	3.22	16.79	6.65	17.62
275	10.58	8.33	2.70	15.14	6.00	15.91
276	9.59	7.32	2.33	13.31	5.24	14.41
277	8.74	6.49	1.95	12.16	4.97	12.83
278	8.35	5.90	1.51	11.02	4.21	11.30
279	8.13	5.17	1.15	10.03	3.58	9.92
280	7.86	4.41	0.76	9.13	3.22	8.69
281	7.66	3.65	0.47	8.55	2.85	7.45
282	7.35	2.77	0.11	7.74	2.34	6.51
283	6.98	2.14	0.07	7.13	1.92	5.82

284	6.73	1.82	0.00	6.42	1.65	5.03
285	6.61	1.56	0.37	5.93	1.56	4.55
286	6.68	1.54	0.88	5.64	1.32	3.88
287	6.76	1.22	0.95	5.33	1.10	3.31
288	6.75	0.94	1.13	4.67	0.86	2.92
289	6.49	0.82	1.26	4.40	0.77	2.46
290	6.39	0.57	1.40	3.80	0.42	2.17
291	6.35	0.47	1.50	3.40	0.13	1.89
292	6.39	0.12	1.40	2.74	-0.33	1.38
293	6.03	0.03	1.79	2.68	-0.49	1.13
294	5.93	0.06	2.03	2.56	-0.62	1.06
295	5.73	0.18	2.18	2.42	-0.51	0.80
296	5.40	0.20	2.29	1.95	-0.80	0.67
297	5.13	0.26	2.38	1.96	-0.71	0.56
298	4.82	0.43	2.50	1.90	-0.65	0.58
299	4.59	0.56	2.60	1.78	-0.46	0.47
300	4.17	0.44	2.57	1.79	-0.52	0.36
301	3.88	0.50	2.43	1.79	-0.48	0.24
302	3.76	0.54	2.40	1.66	-0.49	0.12
303	3.49	0.70	2.58	1.60	-0.38	0.18
304	3.10	0.69	2.33	1.45	-0.49	0.12
305	2.85	0.55	2.19	1.50	-0.52	0.07
306	2.51	0.43	1.94	1.42	-0.58	0.16
307	2.31	0.42	1.94	1.27	-0.71	0.21
308	1.98	0.22	1.57	0.86	-0.96	-0.10
309	1.66	0.18	1.21	0.82	-1.06	-0.16
310	1.49	0.10	1.03	0.67	-1.45	-0.38
311	1.23	-0.05	0.72	0.44	-1.51	-0.54
312	1.11	-0.13	0.34	0.21	-1.62	-0.77
313	0.92	-0.36	0.13	0.06	-1.86	-0.96
314	0.88	-0.58	-0.20	-0.12	-2.06	-1.11
315	0.88	-0.65	-0.46	-0.34	-2.19	-1.05
316	0.77	-0.85	-0.68	-0.69	-2.23	-1.21
317	0.42	-0.84	-0.61	-0.78	-2.15	-1.11
318	0.31	-0.82	-0.64	-0.97	-2.16	-1.11
319	0.16	-0.88	-0.68	-1.14	-2.20	-1.22
320	0.09	-0.88	-0.60	-1.20	-2.03	-1.19
321	-0.01	-0.72	-0.50	-1.13	-1.79	-1.08
322	0.10	-0.63	-0.50	-1.23	-1.76	-1.08
323	0.06	-0.76	-0.50	-1.35	-1.81	-1.15
324	-0.09	-0.77	-0.45	-1.25	-1.69	-1.16
325	-0.04	-0.70	-0.37	-1.23	-1.53	-1.11
326	-0.02	-0.59	-0.11	-1.01	-1.35	-1.05
327	-0.10	-0.43	0.00	-0.84	-1.22	-1.00
328	-0.19	-0.69	-0.13	-0.70	-1.30	-1.10

329	-0.16	-0.82	-0.23	-0.70	-1.27	-1.09
330	-0.14	-0.85	-0.32	-0.72	-1.35	-1.00
331	-0.24	-0.77	-0.33	-0.62	-1.47	-1.00
332	-0.52	-0.88	-0.67	-0.79	-1.73	-1.17
333	-0.49	-0.80	-0.71	-0.52	-1.72	-1.04
334	-0.54	-0.64	-0.67	-0.38	-1.71	-0.83
335	-0.55	-0.66	-0.78	-0.20	-1.66	-0.86
336	-0.42	-0.77	-1.04	-0.20	-1.77	-0.91
337	-0.62	-0.74	-0.93	-0.16	-1.76	-0.84
338	-0.61	-0.56	-1.02	-0.27	-1.67	-0.78
339	-0.54	-0.33	-1.01	-0.30	-1.49	-0.62
340	-0.77	-0.20	-1.00	-0.41	-1.42	-0.68
341	-0.80	-0.03	-0.79	-0.36	-1.35	-0.54
342	-0.76	0.09	-0.76	-0.42	-1.29	-0.43
343	-0.61	-0.01	-0.78	-0.45	-1.32	-0.38
344	-0.57	0.09	-0.65	-0.39	-1.17	-0.22
345	-0.69	0.39	-0.52	-0.36	-0.95	-0.03
346	-0.76	0.44	-0.44	-0.33	-0.87	0.06
347	-0.71	0.42	-0.16	-0.11	-0.63	0.15
348	-0.54	0.49	0.06	0.22	-0.48	0.29
349	-0.44	0.53	0.07	0.32	-0.47	0.25
350	-0.23	0.42	0.07	0.36	-0.49	0.21
351	-0.21	0.34	-0.07	0.39	-0.51	0.18
352	-0.19	0.10	-0.13	0.53	-0.58	0.26
353	0.04	0.01	-0.18	0.44	-0.65	0.24
354	0.07	-0.11	-0.32	0.43	-0.89	0.20
355	0.08	-0.33	-0.46	0.26	-1.11	0.12
356	0.16	-0.53	-0.69	0.09	-1.28	-0.05
357	0.39	-0.68	-0.76	0.04	-1.40	-0.12
358	0.40	-0.62	-1.01	-0.09	-1.55	-0.17
359	0.39	-0.74	-1.08	-0.16	-1.70	-0.40
360	0.43	-0.75	-1.24	-0.29	-1.77	-0.44
361	0.25	-0.73	-1.25	-0.43	-1.78	-0.57
362	0.24	-0.64	-1.39	-0.48	-1.83	-0.56
363	0.14	-0.69	-1.42	-0.56	-1.83	-0.56
364	-0.06	-0.66	-1.45	-0.64	-1.91	-0.53
365	0.12	-0.59	-1.35	-0.63	-1.98	-0.50
366	0.03	-0.62	-1.40	-0.71	-1.99	-0.42
367	-0.06	-0.52	-1.33	-0.74	-2.03	-0.36
368	-0.12	-0.23	-1.16	-0.59	-1.90	-0.23
369	-0.25	0.02	-0.98	-0.47	-1.69	-0.10
370	-0.37	0.03	-0.86	-0.38	-1.68	0.01
371	-0.17	0.06	-0.68	-0.21	-1.62	0.06
372	-0.21	0.17	-0.56	-0.03	-1.61	0.04
373	-0.34	0.18	-0.62	-0.17	-1.65	-0.08

374	-0.36	0.20	-0.50	-0.04	-1.60	0.05
375	-0.51	0.43	-0.38	0.18	-1.48	0.24
376	-0.38	0.24	-0.38	0.21	-1.56	0.19
377	-0.32	0.12	-0.45	-0.02	-1.61	0.02
378	-0.31	-0.02	-0.52	0.00	-1.59	0.04
379	-0.41	-0.08	-0.60	-0.08	-1.49	0.06
380	-0.40	-0.06	-0.68	-0.14	-1.46	0.00
381	-0.42	-0.13	-0.77	-0.27	-1.45	-0.14
382	-0.40	-0.06	-0.92	-0.17	-1.37	-0.13
383	-0.46	-0.12	-0.92	-0.07	-1.26	-0.11
384	-0.34	-0.17	-1.08	-0.12	-1.16	-0.19
385	-0.46	-0.27	-1.17	-0.26	-1.11	-0.34
386	-0.30	-0.31	-1.24	-0.20	-0.93	-0.28
387	-0.32	-0.29	-1.27	-0.07	-0.76	-0.23
388	-0.14	-0.19	-1.20	0.06	-0.61	-0.19
389	-0.04	-0.11	-1.18	0.16	-0.46	-0.15
390	0.06	-0.02	-1.13	0.26	-0.34	-0.11
391	0.14	0.06	-1.07	0.36	-0.24	-0.06
392	0.20	0.14	-0.98	0.45	-0.17	-0.01
393	0.24	0.19	-0.88	0.53	-0.14	0.04
394	0.24	0.23	-0.76	0.59	-0.15	0.08
395	0.21	0.24	-0.62	0.64	-0.21	0.12
396	0.14	0.23	-0.45	0.67	-0.33	0.16
397	0.02	0.17	-0.27	0.67	-0.51	0.18
398	-0.16	0.08	-0.07	0.64	-0.76	0.20
399	-0.39	-0.06	0.16	0.58	-1.08	0.20
400	-0.68	-0.25	0.41	0.49	-1.49	0.19

Table S2. The predicted interactions of 360A-constrained AS1411 to NCL RBD1,2.

Aptamer sites	Interaction (AS1411-NCL)	Interaction (A360-NCL)
T6	LYS125 S	LYS49 S; GLU119 H
G7	LYS49 S	LYS49 S; THR47 H; ARG121 H*2
T9	ARG91 S	ARG91 S
G10		ARG94 S
G11		LYS138 S
T12		LYS138 S
G20	LYS49 S	
Number of interactions	4	10

Notes: H, Hydrogen bond; S, Salt bridge; NCL, nucleolin; RBD1,2, RNA-binding domains 1 and 2.

Table S3. The primers used in the study

Gene name	Forward sequence (5'-3')	Reverse sequence (5'-3')
TNFSF15	CACCACATACCTGCTTGTCAGC	TCTCCGTCTGCTCTAAGAGGTG
PDX1	GAAGTCTACCAAAGCTCACGCG	GGAACTCCTTCTCCAGCTCTAG
CLEC4M	GAGTAACCGCTTCTCCTGGATG	CGCACAGTCTTCATTCCCGCTA
CNR2	AGTGTTGGCTGTGCTCCTCATC	GTTGATGAGGCACAGCATGGAG
CDKN1A	AGGTGGACCTGGAGACTCTCAG	TCCTCTTGGAGAAGATCAGCCG
BAD	CCAACCTCTGGGCAGCACAGC	TTTGCCGCATCTGCGTTGCTGT
CD24	CACGCAGATTTATTCCAGTGAAAC	GACCACGAAGAGACTGGCTGTT
HTR2C	CTAGTGGGACTACTTGTCATGCC	GCGATATAGCGCAGAGGTGCAT
AQP4	GCCATCATTGGAGCAGGAATCC	ACTCAACCAGGAGACCATGACC
SLC25A2	CTGCTTCCTGAAGACATACGCC	CACTTTCCTGACAAACTGCTGGC
NRK	CAGGCTGAAGTCCAGATAGAGC	GTGCATGATCCTGGTTGTTAGGC
IFIT1B	CTTGGCGAAGTGTGGAGGAAAG	TCCAGGCGATAGACGGTGATTG
RFX6	ACTCACTGCCAGTGTATCCTGG	GGAAGGAGATGGTCAGGCATTC
GAPDH	GTCTCCTCTGACTTCAACAGCG	ACCACCCTGTTGCTGTAGCCAA
